# Macrophage membrane (MMs) camouflaged near-infrared (NIR) responsive bone defect area targeting nanocarrier delivery system (BTNDS) for rapid repair: promoting osteogenesis via phototherapy and modulating immunity

**DOI:** 10.1186/s12951-024-02351-5

**Published:** 2024-03-01

**Authors:** Peng Xue, Zhiyong Chang, Hao Chen, Hongzhong Xi, Xiaoxue Tan, Shuai He, Haishi Qiao, Xiaohong Jiang, Xin Liu, Bin Du

**Affiliations:** 1 Department of Orthopedics, Affiliated Hospital of Nanjing University of Chinese Medicine, Hanzhong Road 155, Nanjing, 210029 China; 2 Jiangsu Province Hospital of Chinese Medicine, Nanjing, 210029 China; 3 Department of Pharmaceutical Engineering, School of Engineering, China Pharmaceutical University, Nanjing, 210009 China; 4 International Chinese-Belorussian Scientific Laboratory on Vacuum-Plasma Technology, Nanjing University of Science and Technology, Nanjing, 210094 China

**Keywords:** Bone defect targeting, Black phosphorus nanosheets, Anti-inflammation, Osteogenesis, Phototherapy

## Abstract

Bone defects remain a significant challenge in clinical orthopedics, but no targeted medication can solve these problems. Inspired by inflammatory targeting properties of macrophages, inflammatory microenvironment of bone defects was exploited to develop a multifunctional nanocarrier capable of targeting bone defects and promoting bone regeneration. The avidin-modified black phosphorus nanosheets (BP-Avidin, BP_Avi_) were combined with biotin-modified Icaritin (ICT-Biotin, ICT_Bio_) to synthesize Icaritin (ICT)-loaded black phosphorus nanosheets (BP_ICT_). BP_ICT_ was then coated with macrophage membranes (MMs) to obtain MMs-camouflaged BP_ICT_ (M@BP_ICT_). Herein, MMs allowed BP_ICT_ to target bone defects area, and BP_ICT_ accelerated the release of phosphate ions (PO_4_^3−^) and ICT when exposed to NIR irradiation. PO_4_^3−^ recruited calcium ions (Ca^2+^) from the microenvironment to produce Ca_3_(PO_4_)_2_, and ICT increased the expression of osteogenesis-related proteins. Additionally, M@BP_ICT_ can decrease M1 polarization of macrophage and expression of pro-inflammatory factors to promote osteogenesis. According to the results, M@BP_ICT_ provided bone growth factor and bone repair material, modulated inflammatory microenvironment, and activated osteogenesis-related signaling pathways to promote bone regeneration. PTT could significantly enhance these effects. This strategy not only offers a solution to the challenging problem of drug-targeted delivery in bone defects but also expands the biomedical applications of MMs-camouflaged nanocarriers.

**Graphical Abstract:**

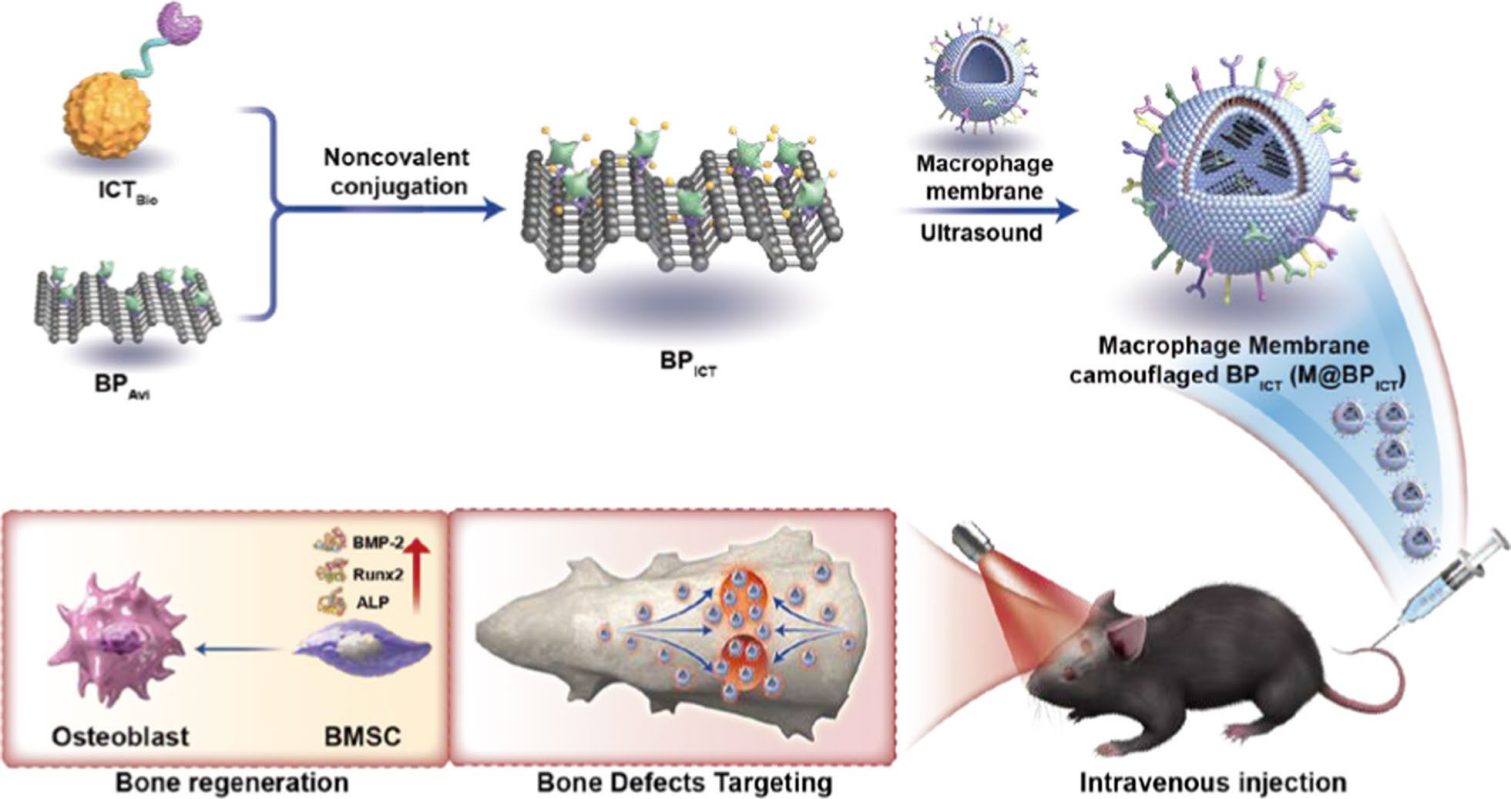

**Supplementary Information:**

The online version contains supplementary material available at 10.1186/s12951-024-02351-5.

## Introduction

Bone defect, a disruption of the structural integrity of bone typically due to trauma, bone tumors, or osteonecrosis, is one of the main cause for non-healing or delayed bone healing [1, 2]. For the bone defect with critical size necessitating surgical intervention, researchers have successfully fabricated a variety of bone repair scaffolds contributed from the development of tissue engineering [3–6]. However, passive self-repair is the only option for patients with non-critical-sized bone defects or patients with bone defects cannot tolerate the surgery. Notably, these patients have comprised the majority of bone defects individuals and been at a high risk of experiencing bone repair failure [7]. Additionally, there are lacking clinically efficient drug delivery system for treatment of bone defect needing self-repair. Therefore, it is urgent to pay close attention to these patients’ requirements for target drug delivery to the bone defect area.

Currently, researchers have designed a variety of bone-targeting nanocarrier systems (BNDS) and demonstrated that these systems are effective at promoting bone regeneration and elevating bone density [8]. Although locally administration of agents is a common routine to manage the bone defect, the systematic administration might be a more appropriate method to treat non-critical-sized bone defect since it is an essentially non-invasive, dosage-regulated bone repair treatment modality. Zheng et al. conjugated alendronate with iron oxide nanoparticles to construct BNDS for treating postmenopausal osteoporosis by intravenous administration exhibiting the regulation effect on local immunity of bone tissues [9]. The targeting properties of BNDS primarily stem from the strong affinity between bisphosphonates, tetracyclines, bone-targeting gene clusters, and hydroxyapatite or osteocytes in bone tissue [10–12]. Thus, although BNDS had an affinity for bone tissues, but it was lacking specificity for bone defect area, which cannot provide adequate drugs for bone defects, ultimately leading to slow or unsuccessful repair. Additionally, BNDS is susceptible to be cleared by immune system leading to a lessened therapeutic effect. Therefore, it is imperative to design a bone defect area targeting nanocarrier delivery system (BTNDS) that can surmount these obstacles.

Previous studies have demonstrated significant microenvironmental changes in bone defect including increased infiltration of inflammatory cells, pH reduction, and a rapid elevation of Ca^2+^ concentration [13]. Consequently, the specific microenvironments of bone defect area might be explored to design BTNDS for the target drug delivery. The immune cells including macrophages and neutrophils or the derived cell membrane had been reported to target the inflammatory microenvironments [14]. The macrophage-derived membranes (MMs) inherit surface ligands or receptors could efficiently target inflammatory sites and absorb the cytokines contributing to the reduction of inflammatory reaction [15]. However, single MMs were not able to serve as an ideal vehicle for drug delivery to inflammatory areas since they were in short of long-term stability and high drug-loading efficiency. Instead, MMs could be utilized to assist other nanocarriers to target inflammatory microenvironments preventing from being cleared and exerting the absorbed performance for inflammatory factors.

Phosphorus (P) is second most abundant mineral in the body and an essential component of natural bone tissues. A two-dimensional (2D) nanomaterial, black phosphorus nanosheets (BP), has attracted tremendous attention in bone repair research due to its excellent biocompatibility and photothermal properties [16–18]. Several studies have reported that BP could serve as drug delivery systems with ultra-high loading capacity and high-efficiency photosensitizers for photothermal therapy (PTT), which could be applied to the repair of bone defect [19, 20]. PTT is a non-invasive, controlled intervention strategy that can promote osteogenesis within an optimal temperature range (40–42 °C) [21, 22]. More importantly, BP could be continuously degraded into nontoxic PO_4_^3−^ and capture Ca^2+^, thereby accelerating biomineralization for the bone regeneration [23, 24]. Shen et al. had designed a bone tissue engineering scaffold doped with BP, which exhibited a preeminent performance on the bone regeneration after combing with photothermal therapy [25]. In another study, multifunctional nanospheres containing BP had been engineered for treatment of osteoporotic fracture upon near-infrared (NIR) irradiation [26]. Therefore, BP is an ideal platform for delivery of bone growth agents and can target the bone defect microenvironments with the introduction of MMs.

**Scheme 1:**
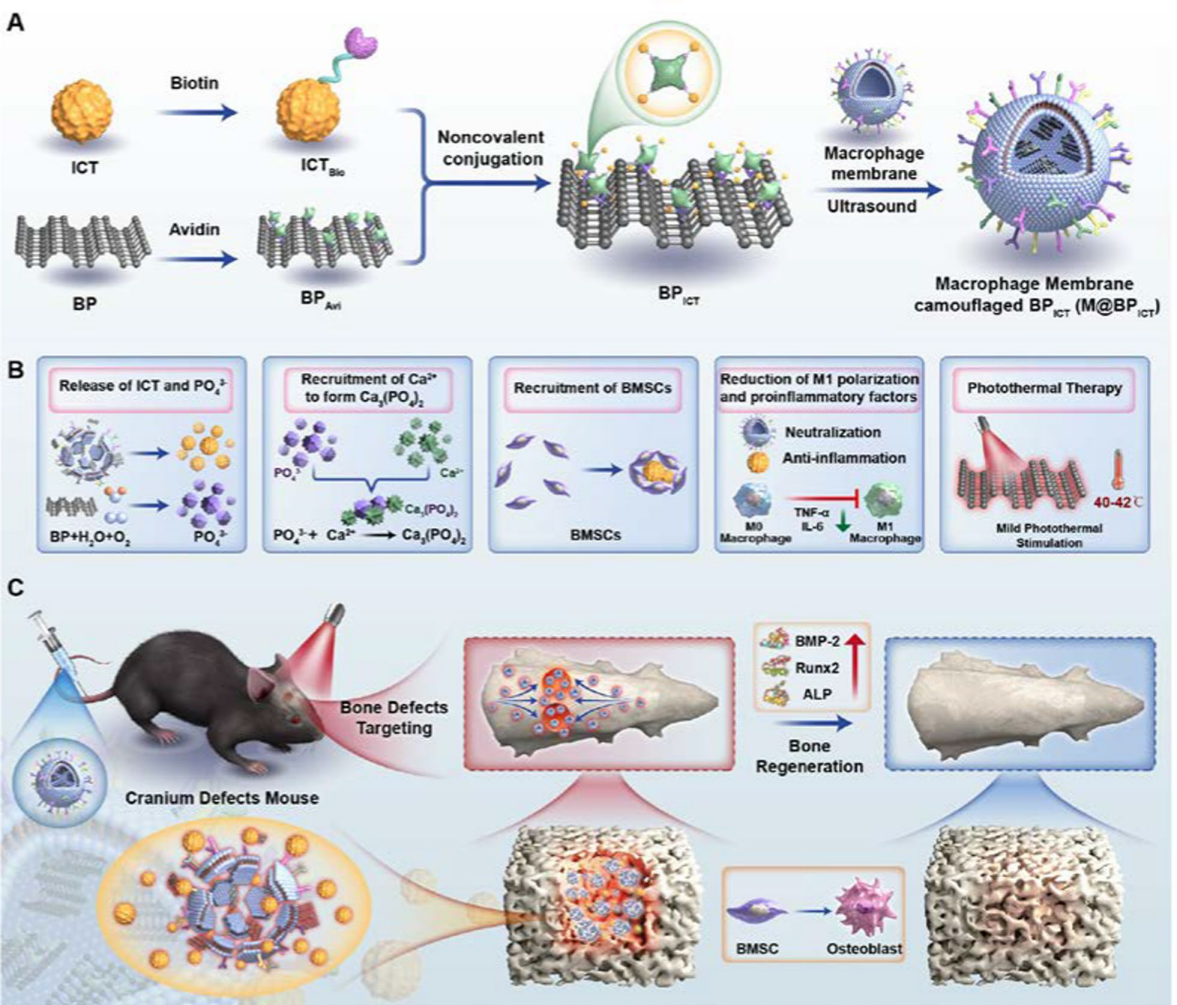
Schematic illustration of the preparation, bone-defect-targeting and multiple bone regeneration mechanisms of M@BP_ICT_. **A** The preparation of M@BP_ICT_. **B** Multiple bone regeneration mechanisms of M@BP_ICT_. **C** The in vivo bone regeneration activity of M@BP_ICT_

In this study, BP had been covalently modified with avidin according to previous report [27]. The avidin-coupled BP (BP_Avi_) could serve as a universal and versatile platform for the efficient delivery of cargos with biotin connection. Here, Icaritin (ICT), a flavonoid monomer isolated from the Chinese herb Epimedium was taken as a modal drug after being modified with biotin. ICT had been reported to inhibit the expression of inflammatory factors and stimulate the proliferation and osteogenic differentiation of bone marrow mesenchymal stem cells (BMSCs) [28]. According to our preliminary research, ICT attached to tissue-engineered scaffolds could promote osteogenic differentiation of BMSCs by increasing the expression of bone morphogenetic protein-2 (BMP-2), Alkaline phosphatase (ALP), and Runt-related transcription factor-2 (Runx2) [29]. The biotin- connected ICT could be efficiently bonded to BP_Avi_ (BP_ICT_) through non-covalent method, which would address the solubility and bioavailability issues for ICT. The MMs was introduced to prepare membrane-encapsulated BP_ICT_ (M@BP_ICT_), which would facilitate the BP_ICT_ with bone defect area targeting capacity. In addition, the MMs could serve as nano-sponges to absorb the inflammatory factors based on the receptors derived from macrophages. After accumulating at the bone defect, the ICT could be released from M@BP_ICT_ exhibiting anti-inflammation and osteogenic activity. More importantly, under the irradiation of NIR, PTT could promote osteogenesis and mediate the degradation of BP into PO_4_^3−^ to accelerate osteogenesis by capturing Ca^2+^. In short, a bone-defect-targeting nano delivery system based on bone repair material and natural compound was engineered providing a promising strategy for self-repair of non-critical-sized bone defects (Scheme 1).

## Materials and methods

### Materials and reagents

Black phosphorus was purchased from Kunming Black Phosphorus Technology Service Co., Ltd. (Kunming, China). Icaritin (ICT), avidin, biotin and lipopolysaccharides (LPS) were purchased from Yuanye Bio-Technology Co., Ltd. (Shanghai, China). Sulfosuccinimidyl4-(*N*-maleimidomethyl) cyclohexa (Sulfo-SMCC), 1-hydroxybenzotriazole (HoBt), *N*,*N*′dicyclohexylcarbodiimide (DCC), *N*,*N*-dimethylformamide (DMF), Dexamethasone, ascorbic acid, dimethyl sulfoxide (DMSO), simulated body fluid (SBF) and β-glycerol Sodium phosphate were purchased from Solarbio Science & Technology Co., Ltd. (Beijing, China). H-MEM and α-MEM and were purchased from Biological Industries, Israel. Phosphate assay kit (ab65622) was purchased from Abcam (Cambridge, UK). Enzyme Linked Immunosorbent Assay (ELISA) kits (IL-6 and TNF-α) were purchased from Multi Sciences Biotechnology Co., Ltd (Hangzhou, China). Fetal Bovine Serum (FBS), Phosphate buffered saline (PBS), Penicillin–streptomycin solution, DAPI solution, Live/Dead staining Kit, ALP staining kit, ARS staining solution, recombinant mouse IL-6 and TNF-α were bought from Beyotime Biotechnology Co., Ltd (Nantong, China). Antibodies (BMP-2, Runx2, ALP and β-Actin) were purchased from Santa Cruz Biotechnology, USA. SweScript All-in-One RT SuperMix for qPCR (One-Step gDNA Remover), 2×Universal Blue SYBR Green qPCR Master Mix and staining kits (H&E, Goldner, BMP-2, CD31, IL-10 and TNF-α) were purchased from Servicebio Technology Co. Ltd (Wuhan, China). Cell culture dishes and cell culture plates were acquired from NEST Biotechnology Co. Ltd (Wuxi, China).

### BP preparation

The BP was obtained by the top-down method of liquid-phase exfoliation from bulk BP. Firstly, BP bulk (500 mg) was put into DMF (30 mL). Next, the BP on the ice bath was performed with an ultrasonic probe (On/Off cycle: 5 s/10 s, Amplifier: 60%) for 8 h. Centrifugation (6000 rpm, 10 min) was used to separate large particles of BP. Afterwards, the liquid supernatant was centrifuged again at 148,000 rpm for 15 min and the precipitate of small particle size of BP was collected.

### BP-avidin (BP_Avi_) preparation

Avidin (1.0 mL, 0.5 mg mL^−1^) and Sulfo-SMCC (660 L, 1.0 mg mL^−1^) were dissolved in 3.34 mL of PBS. The mixture was stirred at 4 °C for 2 h before being dialyzed overnight with a dialysis bag (MWCO: 3500). The obtained product was collected by centrifugation at 14,000×*g* for 10 min to get maleimide-activated avidin. After dispersing BP (1.0 mg) in DMF (2.0 mL), maleimide-activated avidin (1.0 mg) in deionized water (1.0 mL) was added to the BP solution. The reaction was maintained at 30 °C for 8 h in an oil bath with magnetic stirring. The solution was then dialyzed to remove excess maleimide-activated avidin that had not been reacted. After 6 h, the product was collected by centrifuging at 17,000×*g* for 4 min and rinsing three times with deionized water. The BP_Avi_ product was finally stored at 4 °C.

### Synthesis of icaritin-biotin (ICT_Bio_)

Biotin (0.36 g, 1.50 mmol), icaritin (0.15 g, 1.24 mmol), HoBt (0.14 g, 1.00 mmol) and DMAP (0.035 g, 0.10 mmol) were dissolved in 10 mL of DMF. The mixture was stirred for 1 h at room temperature before being cooled to 0 °C in an ice bath. DCC (0.21 g, 1.00 mmol) was dissolved in 10 mL of DMF and then dropwisely added to the the above mixture. After removing the ice bath, the reaction was gradually warmed to room temperature and continued for 24 h. The crude mixture was filtered and rinsed with CHCl_3_ prior to vacuum-assisted drying. The dried product was resuspended and dissolved in 20 mL absolute ethanol, heated to 80 °C prior to filtration, and the filter cake was rinsed 2 to 3 times with hot ethanol before being dried to yield 0.26 g of white powder biotin-icartin (31.7%). The structure was verified by ^1^H NMR (Bruker ECX 500, Germany).

### BP-icaritin (BP_ICT_) preparation

Briefly, ICT_Bio_ (10 mg) dissolved in DMF (5 mL) was added with BP_Avi_ (10 mg). In addition, the reaction was maintained for 2 h at room temperature, followed by dialysis to remove excess unreacted biotin-icaritin. The product was collected by centrifuging at 17,000×*g* for 4 min and rinsing three times with deionized water. The final product was then stored at 4 °C.

### Macrophage membranes (MMs) collection

MMs were collected according to previous study [30]. Briefly, the macrophages were digested with trypsin, centrifuged at 1000 rpm for 5 min, the supernatant was discarded, and the cells were rinsed with PBS. The reagent for cell lysis (pH 7.4) was consisted of Tris (0.01 M), MgCl_2_ (1 mM), phenylmethylsulfonyl fluoride (PMSF, 2 mM), sucrose (0.35 M), and DNase (10 g mL^−1^). Cells were incubated with cell lysate buffer, homogenised at 22,000 rpm for 2 min, and centrifuged at 4 °C, 4000 rpm for 10 min. The supernatant was collected and centrifuged at 4 °C, 10,000 rpm for 20 min. The supernatant was then discarded, and the precipitate was collected following three times of washing with the lysate buffer and dialysis to remove various buffer salts. Finally, the purified MMs were storage as a freeze-drying precipitate.

### Preparation of M@BP_ICT_

MMs (2 mg) were re-dissolved in PBS (1 mL) by the vortex. BP_ICT_ (1 mg) was dispersed in 1 mL of PBS in a water bath. To generate M@BP_ICT_, the MMs solution was slowly dropped into 100 μL of BP_ICT_ solution under ultrasonic conditions for 5 min. The final product was then stored at 4 °C.

### Characterization of nanomaterials

FTIR spectra of BP, ICT, ICT_Bio_, BP_Avi_ and BP_ICT_ were obtained on the Fourier Transform Infrared Spectrometer (FTIR, Thermo Fisher Scientific, USA). Thermogravimetric analysis (TGA, TA Instruments, USA) was performed on a thermos analyzer. Scanning Electron Microscope (SEM, Thermo Fisher Scientific, USA) and Atomic Force Microscope (AFM, Bruker, German) were conducted to analyze the morphology of BP, BP_Avi_ and BP_ICT_. Elemental Mapping analyses of BP_Avi_ and BP_ICT_ were performed using the Transmission Electron Microscope/Energy dispersive X-Ray Spectroscopy (TEM-EDS, FEI talos F200x G2, USA). The size distribution of BP, BP_Avi_, BP_ICT_ and M@BP_ICT_ were characterized by Dynamic Light Scattering (DLS, Wyatt Technology, USA) and Transmission Electron Microscope (TEM, FEI Philips Tecnai 20, USA). The Zeta potential of BP, ICT_Bio_, BP_Avi_ and BP_ICT_ were obtained by DLS.

### Characterization of membrane proteins

MMs and M@BP_ICT_ (at the same MMs concentration of 0.5 mg mL^−1^) were fractionated by 10% sodium dodecyl sulfate polyacrylamide gel electrophoresis (SDS-PAGE). Protein image was obtained after coomassie brilliant blue staining. The cell membrane surface proteins, including interleukin-6 receptor (IL6-R) and tumor necrosis factor receptor type 1 (TNF-R) were detected by western blot assay.

### Evaluation of cytokine neutralization

MMs (1 mg mL^−1^) in different polarization states (M0, M1 and M2) were utilized to encapsulate the BP_ICT_ and t were incubated with PBS containing 10% FBS and TNF-α (1000 pg mL^−1^) or IL-6 (1000 pg mL^−1^) at 37 °C for 1 h. Samples were then centrifuged at 10,000 rpm at 4 °C to remove the nanomaterials, and the concentration of cytokines in the supernatant were measured by ELISA kits (Multi Sciences, China).

### In vitro photothermal measurement

The temperature changes of BP_ICT_ and M@BP_ICT_ upon NIR irradiation were monitored using a visual thermal imaging camera (FLIR, USA). Briefly, 300 μL of BP_ICT_ or M@BP_ICT_ suspension (2.0 mg mL^−1^ BP_ICT_) was respectively exposed to NIR irradiation (808 nm, 1.0 W cm^−2^, 10 min). The photo-thermal images were captured continuously at various time intervals.

### Photothermal conversion efficiency measurement

The photothermal conversion efficiency can be used to evaluate the photothermal conversion performance of nanomaterials. By continuously heating a suspension of water at a certain concentration under laser irradiation until a stable temperature is reached, then switching off the laser and recording the temperature–time curve during the cooling process. The photothermal conversion efficiency can be calculated based on this cooling curve using a specific formula according to the literature [31].

### In vitro release of ICT

Under various pH conditions, the ICT released from M@BP_ICT_ was investigated in PBS with or without NIR irradiation (808 nm, 1.0 W cm^−2^). Two copies of M@BP_ICT_ were placed in dialysis tubes (MWCO: 3500) and immersed in 20 mL of release medium with different pH conditions (pH 7.4 and pH 4.5) while stirring at 37 °C. M@BP_ICT_ samples were irradiated for 10 min by NIR at predetermined intervals, and then returned back into the dialysis tube and immersed into the release medium. At the specified intervals, 1 mL of the release medium was collected and replaced with new medium. The amount of released ICT was measured by the High Performance Liquid Chromatography (HPLC, Agilent Technologies Co. USA) and calculated according to an ICT standard curve. Release experiments were conducted three times, and the results were presented as the mean standard deviation (SD).

### In vitro degradation of BP_ICT_

The different formulations of BP (1 mg) was placed in an EP tube containing 1.5 mL of SBF. The NIR irradiation was performed following condition: 808 nm at 1.0 cm^−2^, 10 min per day, and the other groups without NIR irradiation were placed at room temperature for 7 days. Elemental mapping analysis (C, O, and Ca) were carried out on the elemental analyser, and the presence of Ca_3_(PO_4_)_2_ was analyzed by X-ray diffraction (XRD, Shimadzu, Japan). Experiments were performed according to experimental methods in the literature [32]. The release of PO_4_^3−^ from BP_ICT_ was determined using a phosphate assay kit (ab65622, Abcam, Cambridge, UK) to measure the concentration of PO_4_^3−^ in the double-distilled water (ddH_2_O). The absorbance at 650 nm wavelength was read on a UV–Vis absorbance microplate reader (Thermo Fisher Scientific, USA), and the concentration of PO_4_^3−^ was determined using the standard curve method. 10 mL BP_ICT_ solution (1486.8 μg L^−1^, calculated by 1 μM ICT) was placed in an EP tube, and before each measurement, it was centrifuged, and 10 mL of the supernatant was taken for PO_4_^3−^ content measurement. Subsequently, 10 mL of ddH_2_O was added to the original EP tube to continue immersing BP_ICT_ in a 37 °C constant temperature chamber. When calculating the degradation rate, the total PO_4_^3−^ content was quantified based on the total amount of BP_ICT_ added to the test tube. Additionally, the BP_ICT_+NIR group required additional NIR irradiation (808 nm, 1.0 W cm^−2^, 10 min per day).

### Cells and cell culture

BMSCs and Raw 264.7 were purchased from National Collection of Authenticated Cell Cultures (Shanghai, China). BMSCs were cultured in α-MEM complete medium (10% FBS, 1% penicillin–streptomycin solution), and RAW264.7 cells were in H-DMEM complete medium (10% FBS, 1% penicillin–streptomycin solution). Cells were incubated in a constant temperature containing 5% CO_2_ incubator at 37 °C (Shanghai Jing Hong Co, China). Medium was changed every 2 days. Cells were passaged upon 80–90% confluence under the microscope (Olympus, Japan), and P3 generation cells were obtained for subsequent experiments.

Unless otherwise specified, the dosages for the cellular experiments were determined based on the effectiveness of 1 μM ICT in promoting BMSCs proliferation and osteogenic differentiation (Additional file 1: Figs. S1 and S2): ICT (368.4 μg L^−1^), BP_Avi_ (1118.4 μg L^−1^), BP_ICT_ (1486.8 μg L^−1^), M@BP_Avi_ (1118.4 μg L^−1^), M@BP_ICT_ (1486.8 μg L^−1^), LPS (100 ng mL^−1^) and the NIR irradiation condition: 808 nm, 1.0 W cm^−2^, 10 min per day. Notably, the concentration of BP used in this study is significantly lower than 50 μg mL^−1^, therefore it would not pose cellular toxicity [33].

### In vitro cellular uptake assays

BMSCs (2.5 × 10^4^ cells/well) were seeded in a 24-well plate with fibronectin-coated glass slides. After 12 h incubation, the cells were incubated with Cy5.5-modified M@BP_ICT_ (BP_ICT_: 500 μg mL^−1^, calculated by BP) for different time. The Cy5.5-modified M@BP_ICT_ was prepared by the reaction of the NHS-ester on Cy5.5 with the hydroxyl group of the ICT on BP_ICT_. Briefly, Cy5.5-NHS (2 mg, dissolved in 1 mL DMSO) was added slowly dropwise to BP_ICT_ (50 mg, dispersed in 5 mL PBS), and reacted at room temperature in the dark for 6 h. After that, unreacted Cy5.5-NHS was removed by dialysis in deionized water. The culture medium was discarded and the cells were rinsed twice with PBS and then fixed with 4% paraformaldehyde for 15 min. DAPI (5 μg mL^−1^) was used to stain the cell nucleus at room temperature for 10 min. The slides were scanned with CLSM and analyzed by the ZEN software (ZEISS, Germany). The cellular uptake of nanoparticles was further quantified by flow cytometry (FCM). Briefly, BMSCs were cultured in a 24-well plate (1.0 × 10^5^ cells/well) overnight followed by the treatments as mentioned above. After discarding the culture medium, the cells were rinsed thrice with PBS and treated with trypsin. The cell suspensions were washed twice with PBS, re-suspended in PBS and analyzed by FCM.

### Cytotoxicity analysis

Cell proliferation was measured with CCK8 assay. Briefly, BMSCs were seeded and co-cultured with different nanomaterials in 96-well plates at a density of 3 × 10^3^ cells/well. At 24 h, 48 h, 72 h and 120 h, 10 μL of CCK8 reagent (100 μL medium/well) was added and incubated with cells for 2 h at 37 °C, 5% CO_2_. The absorbance of each well at 450 nm was measured with an automatic enzyme instrument (Bio Tek, USA). Live/Dead staining was conducted according to the manufacturer’s instructions after cells were cultured as above. Finally, dead cells were stained red, and live cells were stained green. Fluorescence images were captured using an inverted fluorescence microscope (Olympus, Japan).

### Cell scratch test

The effects of the various nanomaterials on cell migration in vitro were evaluated using a wound healing assay. BMSCs were cultured in 6-well plates until they attained at least 90% confluence for the scratch tests. A 200 μL sterile pipette tip was used to scratch the BMSCs monolayer after starvation for 24 h. Subsequently, the original medium was replaced by BP_ICT_ or M@BP_ICT_ complete medium. Finally, an inverted microscope (Olympus, Japan) was used to record the migration positions at 24 h. The migration area was calculated using ImageJ software (NIH, Bethesda, MD, USA).

### In vitro osteogenesis

#### Osteogenic induction under inflammatory-free microenvironment

The BMSCs were seeded at 2.5 × 10^4^ cells per well in 12-well plates in the complete medium for 24 h. Then replacing the original medium with osteogenic induction medium (50 μg mL^−1^ ascorbic acid, 10 mM β-glycerol Sodium phosphate and 100 nM dexamethasone were added into the complete medium) with the presence of BP_Avi_, BP_ICT_ and M@BP_ICT_, respectively and cultured for 14 and 21 days. Typically, one group was cultured without the NIR irradiation. The other group was irradiated with NIR, 10 min per day. Osteogenic induction medium was changed every 2 days. ALP and alizarin red S (ARS) staining was then performed at days 14 and 21 of osteogenic induction, respectively. Furthermore, western blotting was conducted at 7 days of osteogenic induction.

#### Osteogenic induction under inflammatory microenvironment

The osteogenic induction medium under inflammatory microenvironment was prepared according to the literature [34]: Raw 264.7 cells were seeded in 12-well plates at a density of 5 × 10^5^ per well. The culture medium was collected from the control, LPS, LPS+BP_ICT_, LPS+M@BP_ICT_ and LPS+M@BP_ICT_+NIR after 24 h co-cultured. After centrifugation, the supernatant was mixed at a 1:2 ratio by volume with osteogenic induction medium to obtain osteogenic induction medium under inflammatory microenvironment. According to the different types of Raw 264.7 culture medium in configuring the osteogenic induction medium under inflammatory microenvironment, it was divided into the following groups: control, LPS, LPS+BP_ICT_, LPS+M@BP_ICT,_ and LPS+M@BP_ICT_+NIR groups. The rest of the steps were as described above. After 14 days of osteogenic induction, ALP staining was conducted to evaluate the osteogenic differentiation level.

### ALP and ARS staining

For each predetermined point, ALP staining was used to assess osteogenesis capacity of each group. Briefly, the supernatant was removed and the cells were rinsed with PBS followed by the addition of 4% paraformaldehyde for 5 min, then rinsed with distilled water. Next, the cells were incubated with ALP staining solution for 20 min. The blue areas in an inverted fluorescence microscope could be interpreted as positive areas. The pre-processing steps of ARS staining were described as above, and calcium nodule formation can be seen as orange-red in an inverted fluorescence microscope (Olympus, Japan) and was interpreted as a positive area using Image J software.

### Flow cytometry

An analysis of the abundance of the M1 marker CD86 was carried out by flow cytometry. Following 48 h of culture, cells were collected, washed, blocked for 15 min with blocking buffer, and then stained for 1 h with PE-conjugated anti-CD86 antibodies. Then the cells were centrifuged to remove extra antibodies and rinsed with PBS. Finally, the cells were analyzed using a flow cytometer and analyzed with FlowJo software.

### ELISA

Raw 264.7 cells were seeded in 12-well plates at a density of 5 × 10^5^ per well. The culture medium was collected from the control, LPS, LPS+BP_ICT_, LPS+M@BP_ICT_ and LPS+M@BP_ICT_+NIR after 48 h co-cultured. After centrifugation, the supernatant was collected. Cytokines (TNF-α and IL-6) in the supernatant were measured using an ELISA kit according to the manufacturer’s instructions.

### Western blotting

After osteogenic induction for 7 days, cells were collected and lysed in RIPA buffer. BCA protein assay kit was used to measure the concentration of protein samples. Then, proteins were separated by SDS-PAGE and transferred to PVDF membrane. The membrane was blocked with rapid blocking solution for 15 min before being incubated with relevant antibodies (BMP-2, Runx2, ALP and β-Actin), followed by the second antibody. Finally, the target protein fragment was detected using a chemiluminescence image analyzer (BIO-RAD, USA), and the grey scale of protein bands was quantified by Image J software.

### RNA sequencing (RNA-seq) and qPCR analysis

#### RNA-seq

Cell lysis was performed using Trizol reagent, and the cell lysate was stored at − 80 °C prior to sequencing. The Illumina Novaseq™ 6000 platform (LC Bio Technology CO., Ltd., Hangzhou, China) was used for paired-end sequencing with a sequencing mode of PE150. Subsequently, the sequencing data was aligned to the human genome (Homo sapiens, GRCh38) using HISAT2 software (https://ccb.jhu.edu/software/hisat2). Assembly of genes or transcripts and FPKM quantification were performed using StringTie software (https://ccb.jhu.edu/software/hisat2). Differential analysis was carried out using the R package edgeR (https://bioconductor.org/packages/release/bioc/html/edgeR.html), with a fold change > 2 and a p-value < 0.05. Finally, GO and KEGG pathway enrichment analysis were performed using DAVID software (https://david.ncifcrf.gov/). The RNA sequencing and analysis were conducted with the assistance of LC Bio Technology CO., Ltd.

#### qPCR

Total RNA was extracted from the cells and reverse transcribed into cDNA for real-time qPCR analysis. The primers for the target genes were synthesized by Wuhan Servicebio Technology Co., Ltd, and all the sequences are shown in Additional file 1: Table S1. 2^−ΔΔCт^ method was used to normalize the gene expression.

### Animal handing

The study was approved by the Ethics Committee of Affiliated Hospital of Nanjing University of Chinese Medicine (Approval No: 2021 DW-11-02). Six-week-old male C57BL/6 mice weighing 19–21 g were randomly divided into six groups (n = 12). All mice received humane care according to the guideline of the Guidebook for the Care and Use of Laboratory Animals. A traditional murine cranium defects model was applied in this study [35]. First, the mice were anesthetized with isoflurane. A non-critical sized circular defect (*Φ*3 mm with full-thickness) was then made on the cranium of each mouse by a trephine. The bone defects repair experiments were conducted by injecting different nanomaterials through the tail vein, and PBS group was used as control. After the mice were sacrificed at week 2 and week 4, the craniums and major organs (heart, liver, spleen, lung and kidney) were harvested. Part of the craniums were further immersed in liquid nitrogen, and the rest samples were stored in the paraformaldehyde solution.

Unless otherwise specified, the following dosages calculated by 8 mg kg^−1^ of ICT were adopted in the animal experiments: PBS (100 μL), M@BP_Avi_ (24.3 mg kg^−1^), M@BP_ICT_ (32.3 mg kg^−1^), M@BP_ICT_ (32.3 mg kg^−1^). Each group of mice with bone defects received a tail vein injection of 100 μL of the formulations once a week. For the groups involving NIR irradiation, the bone defects area was irradiated with NIR irradiation (808 nm, 1.0 W cm^−2^, 10 min) under anesthesia 6 h after the administration.

### In vivo biodistribution

In vivo biodistribution of M@BP_ICT-Cy5.5_ was demonstrated using the C57BL/6 mice bearing cranium injury. The mouse with cranium injury model was established by drilling hole with a handheld grinding drill. After 3 days, the skull injury mice were randomly grouped and injected with a single dose of Cy5.5-modified BP_ICT_ and M@BP_ICT_ (BP_ICT_: 32.29 mg kg^−1^) in 100 μL of PBS via tail vein. Cy5.5-modified BP_ICT_ was prepared by the reaction of the NHS-ester on Cy5.5 with the hydroxyl group of the Tris on ICT. Briefly, Cy5.5-NHS (2 mg, dissolved in 1 mL DMSO) was slowly dropped into BP_ICT_ (50 mg, dispersed in 5 mL PBS), and reacted at room temperature in the dark for 6 h. After that, unreacted Cy5.5-NHS was removed by dialysis in deionized water. At designated time intervals (0, 1, 2, 4, 8, 12 h) post-injection, the fluorescence images were acquired. At the end of experiments, the major organs were dissociated after euthanasia for ex vivo fluorescence imaging. The fluorescence images were scanned using a near-infrared fluorescence imaging system at an excitation of 675 nm and emission of 694 nm, and the images were acquired and analyzed using the Living Image software (PerkinElmer, USA).

### In vivo photothermal measurement

The mice received intravenous administration of PBS, BP_ICT_ and M@BP_ICT_ respectively. Six hours later, the cranium defects area in the mice under anesthesia was irradiated with NIR irradiation (808 nm, 1.0 W cm^−2^, 15 min). A thermal imaging camera (FLIR, USA) was utilized to capture the photo-thermal images and the corresponding temperature variation was monitored.

### Micro-CT

The new bone formation and bone quality of cranium defects area were evaluated by micro-CT (Hiscan, China). The scan parameters were set as 80 kV, 500 μA current, and 25.0 μm pixels. After 3D reconstruction, bone mineral density (BMD), trabecular number (Tb. N), trabecular thickness (Tb. Th) and bone tissue volume/total tissue volume (BV/TV) were measured and recoded by Hiscan Analyzer software (V3.0).

### Histological staining

Craniums were completely decalcified in 10% EDTA and dehydrated in a graded ethanol series, and then embedded in paraffin. The specimens were cut into 5 μm thick sections for histological and immunohistochemical analysis. According to the instructions, H&E, Goldner, BMP-2, CD31, IL-10 and TNF-α staining were performed. An optical microscope (Nikon, Japan) was used to analyze the results. Image J software was used to assess the integrated optical density (IOD) value of the positive area.

### In vivo biosafety analysis

Major organs, including hearts, livers, spleens, lungs, and kidneys, were excised, fixed with 4% paraformaldehyde and sectioned for H&E staining. Besides, the weight of mice at observed time points (0 d, 2 d, 5 d, 7 d, 14 d, 21 d and 28 d) had been recorded.

### Statistical analysis

All data were presented as Mean ± SD. ANOVA with a Tukey post-hoc test was used to conduct data analysis between each group unless indicated otherwise. *P < 0.05, **P < 0.01, ***P < 0.001 or ^#^*P* < 0.05, ^##^*P* < 0.01, ^###^*P* < 0.001 were considered significant. Quantifications were done with Image J software on high-resolution images. Statistical analysis was performed using SPSS 22.0 Software (IBM, USA).

## Results and discussion

### Fabrication and characterization of M@BP_ICT_

**Fig. 1:**
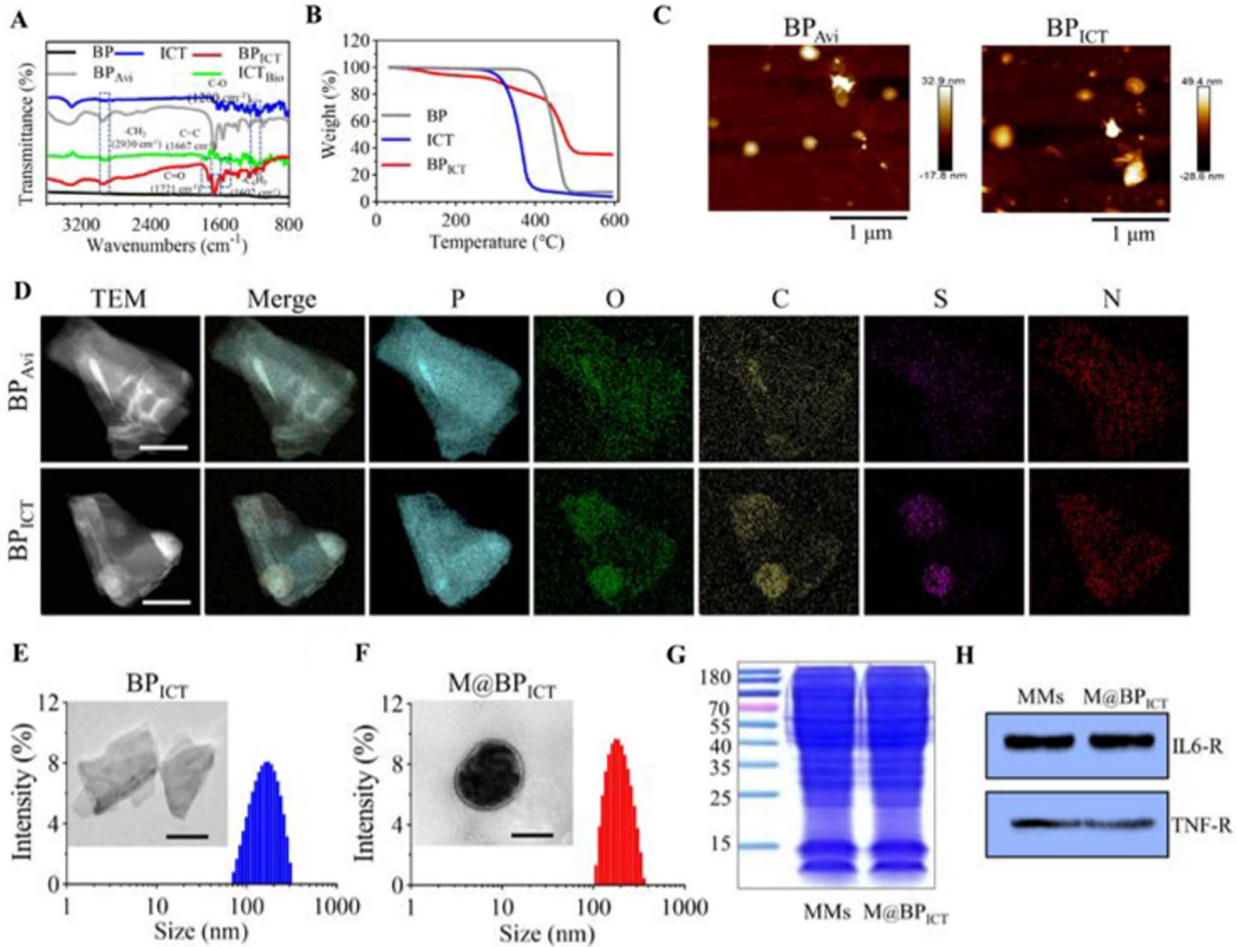
Preparation and characterization of M@BP_ICT_. **A** The FTIR spectra of BP, ICT, ICT_Bio_, BP_Avi_ and BP_ICT_. **B** Thermogravimetric analysis of BP, ICT and BP_ICT_. **C** The AFM images of BP_Avi_ and BP_ICT_. **D** The elemental mapping images of BP_Avi_ and BP_ICT_. Scale bars: 50 nm. **E**, **F** The diameter distribution of BP_ICT_ (**E**) and M@BP_ICT_ (**F**) was measured by DLS and TEM. Scale bars: 100 nm. **G** Protein profiles of macrophage membrane (MMs) and M@BP_ICT_ with Coomassie brilliant blue staining. **H** Expression of IL6-R and TNF-R in MMs and M@BP_ICT_ by western blot analysis

The naked BP was prepared by the method of “top-down” liquid-phase mechanical exfoliation [30, 36]. Then, biotin-coupled ICT was synthesized and confirmed by ^1^H NMR before being connected to BP (Additional file 1: Figs. S3 and S4). To facilitate the BP with high loading efficiency for biotin-coupled ICT, avidin was covalently modified to the surface of BP (Additional file 1: Fig. S5), which could serve as a universal and versatile platform for drug delivery. Accordingly, biotin-coupled ICT could be efficiently linked with BP_Avi_ via non-covalent interaction, which was confirmed by FTIR assay (Fig. 1A). As shown in Fig. 1A, the successful modification of BP and avidin was demonstrated by the presence of characteristic peaks in BP_Avi_, such as the N–H stretching vibration peak at 3312 cm^−1^. Additionally, peaks at 1602 and 2930 cm^−1^ indicated the presence of the phenyl ring and methylene group, respectively. Peaks at 1721 and 1200 cm^−1^ confirmed the successful coupling of ICT and biotin. The carbon–carbon double bond exhibited a peak at 1667 cm^−1^. Detection of these characteristic peaks in BP_ICT_ indicated the ICT was successfully connected to the BP. Additional file 1: Fig. S6 indicated that XRD of BP exhibits prominent characteristic peaks at 16.9°, 26.6°, 34.3°, and 52.4°. The intensities and positions of the diffraction peaks are in basic agreement with BP’s crystal database standard card (JCPDS No. 47-1626), corresponding to the (020), (021), (040), and (060) crystal planes of BP. This indicated that the BP prepared by the liquid exfoliation method have high purity and demonstrates the stable and excellent crystal structure of the obtained BP. Furthermore, the diffraction peaks of BP_ICT_ are highly consistent with BP, indicating that the surface modification of the compound does not affect its crystalline characteristics. Furthermore, TGA was performed to calculate the drug-loading capacity of BP. By analyzing the mass difference corresponding to the weight loss stages of BP and ICT on the BP_ICT_ TGA curve, the mass ratio of ICT to BP in BP_ICT_ is determined to be 2:5. (Fig. 1B). The AFM revealed that the thicknesses of BP (Additional file 1: Fig. S7), BP_Avi_, and BP_ICT_ were 25.1, 32.9, and 49.4 nm, respectively (Fig. 1C) and thickness elevation might be attributed to the surface modification. Furthermore, the SEM results demonstrated that different formulations of BP were well maintained as a nanosheet structure (Additional file 1: Fig. S8). The modification of ICT on BP was confirmed by element mapping demonstrating that the increased carbon and oxygen elements (main elements of ICT) were detected on BP (Fig. 1D). Furthermore, the presence of sulfur and nitrogen elements (specific elements of avidin and biotin) on BP indicated that ICT was connected to BP through the interaction of avidin and biotin. Theoretically, S should not be present on BP_Avi_, but the little presented S element may be due to the use of S-containing sulfo-SMCC during the synthesis of BP_Avi_ (Additional file 1: Fig. S9). Additionally, due to the successful loading of ICT on the material, the C and O elements in BP_ICT_ have increased by 9.2% and 3.49% respectively compared to BP_Avi_. The above studies demonstrated the ICT was successfully coupled with BP to form BP_ICT_ through the non-covalent conjunction of avidin and biotin. The acute phase of bone defects is always accompanied with the secretion of excessive pro-inflammatory factors, which can significantly inhibit the osteogenic differentiation of BMSCs [37]. Instead, inflammatory microenvironments could be targeted by inflammatory cells or the derived membrane. The cell membranes extracted from macrophages were employed to encapsulate the BP_ICT_ to endow it with bone-defect-targeting ability. Previous research indicates that small-sized BP (with a mean diameter of 208.5 ± 46.9 nm) demonstrates better biocompatibility [38]. Subsequently, the size and morphology were investigated by DLS and TEM. As shown in Fig. 1E, F, the average diameter of BP_ICT_ and MMs-encapsulated BP_ICT_ (M@BP_ICT_) was about 174 nm and 198 nm, respectively, which was significantly larger than naked or avidin-modified BP (Additional file 1: Fig. S10). Therefore, these nanomaterials could exhibit good biocompatibility. Additional file 1: Fig. S11 demonstrated that due to the presence of oxidized phosphoric groups on the surface of BP, the surface potential of exposed BP is relatively negative at − 37.9 mV. Following the binding of the avidin, the surface potential increases to − 27 mV due to the positively charged avidin neutralizing some of the negative charges under neutral pH conditions. Subsequently, upon binding with ICT_Bio_, the surface potential further increases to − 20.6 mV. The negative charge is advantageous for the long circulation of biomimetic BP nanocomposites in vivo, avoiding clearance by the body’s immune system, particularly the reticuloendothelial system. Furthermore, M@BP_ICT_ were characterized with obvious bilayer membrane structure revealed by the TEM image. The similarity of Coomassie brilliant blue staining indicated that the membrane proteins of the MMs were retained in ultrasound-prepared M@BP_ICT_ (Fig. 1G). Moreover, western blot assay demonstrated that IL-6R and TNF-R protein could be detected in both MMs and M@BP_ICT_ (Fig. 1H), indicating that M@BP_ICT_ might decrease inflammatory reaction by adsorbing pro-inflammatory factors in the bone defects area.

### Evaluation of photothermal property and degradation processes

**Fig. 2:**
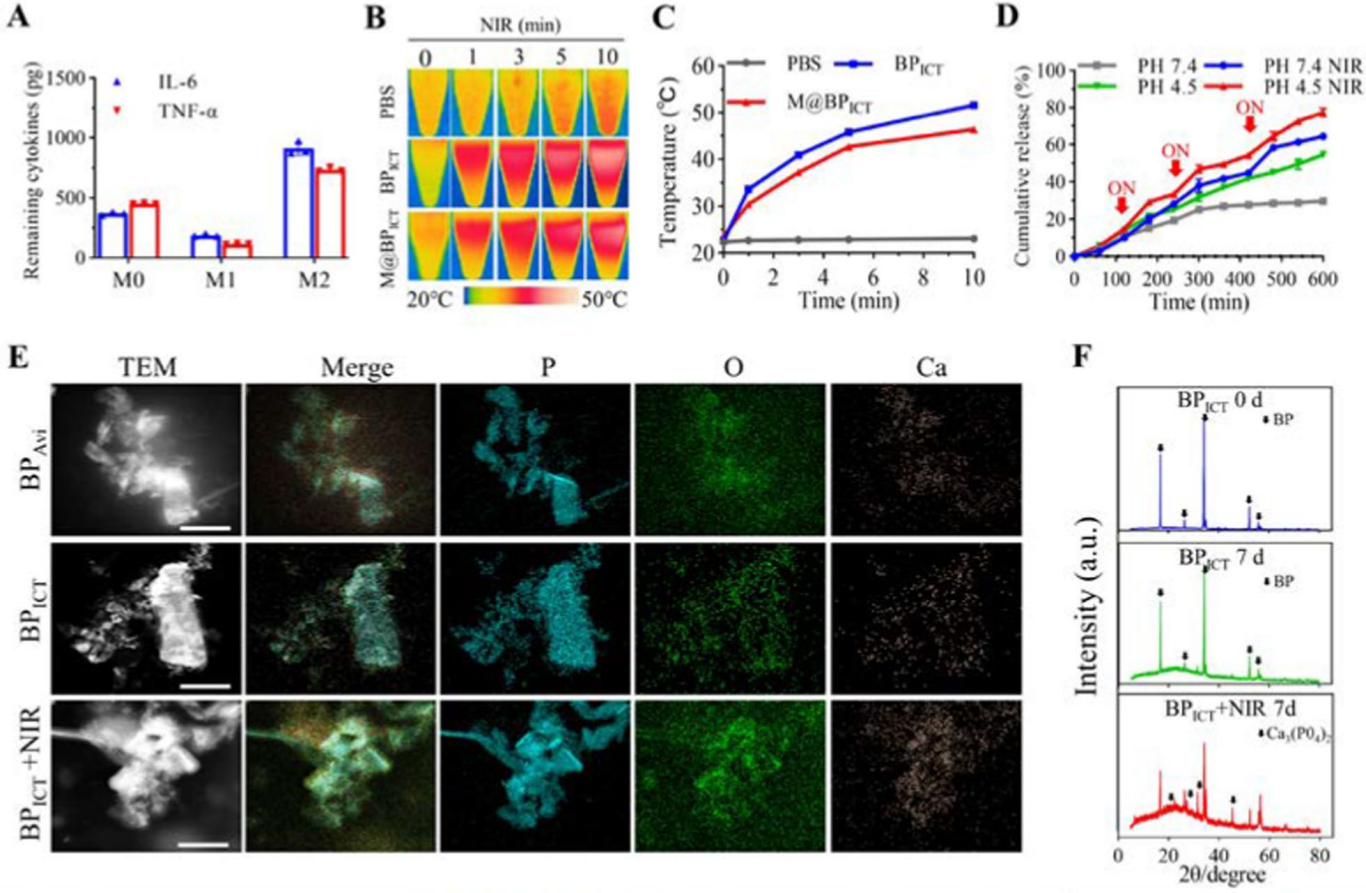
The functional analysis for M@BP_ICT_. **A** The inflammatory factors adsorption of the M@BP_ICT_ with different sources of cell membrane (M0, M1 and M2 macrophages). **B** Photothermal investigation for the different formulations of BP exposed to different time of NIR irradiation (808 nm, 1.0 W cm^−2^). **C** Temperature measurement for different formulations of BP exposed to varied time of NIR irradiation (808 nm, 1.0 W cm^−2^). BP_ICT_ or M@BP_ICT_ suspension contained BP_ICT_ at a concentration of 1486.8 μg L^−1^ calculated by 1 μM ICT. **D** The cumulative release of ICT from BP_ICT_ with or without NIR irradiation (808 nm, 1.0 W cm^−2^, 10 min) at different pH conditions. **E** TEM elemental mapping for BP_Avi_, BP_ICT_ and BP_ICT_+NIR in SBF for 7 d. NIR irradiation: 808 nm, 1.0 W cm^−2^, 10 min per day. Scale bars: 50 nm. **F** XRD pattern of BP_ICT_ after incubation in SBF for 0 day and 7 days. NIR irradiation condition: 808 nm, 1.0 W cm^−2^,10 min per day. The black arrows indicated Ca_3_(PO_4_)_2_

The MMs inherited the membrane from macrophages, which might be advantageous to the adsorption of inflammatory factors. Therefore, the adsorption capacity of M@BP_ICT_ for inflammatory cytokines was evaluated by ELISA. As shown in Fig. 2A, both of the membranes from M0 and M1 macrophages could absorb inflammatory factors of IL-6 and TNF-α through the cytokine receptors on the membrane. In contrast, the cell membranes from the anti-inflammatory M2 macrophages did not exhibit significant absorbed effect. Furthermore, the photothermal performance of M@BP_ICT_ was studied by monitoring the temperature variation upon different period of NIR irradiation. The results demonstrated the photothermal characteristic of BP was well maintained after the chemical modification and MMs encapsulation indicated by the elevated temperature as the NIR-exposure time extended. The temperature of M@BP_ICT_ could reach up to 46.4 °C after 10 min NIR irradiation (Fig. 2B, C). BP could serve as a stable and efficient drug delivery system under the chemical modification and interaction of biotin and avidin. Previous studies have demonstrated that the cargos delivered by BP could be released in response to acid microenvironments and NIR irradiation since both of the conditions could accelerate the degradation of BP [36]. According to the published method [31], under 808 nm laser irradiation, BP_ICT_ and M@BP_ICT_ demonstrated a photothermal conversion efficiency (η) of 27.3% and 26.1% respectively (Additional file 1: Fig. S12). Next, the release of ICT from BP under different pH conditions and NIR exposure was studied. As shown in Fig. 2D, the acid condition or NIR exposure could accelerate the ICT release from BP compared with the condition of pH7.4. However, the combination of NIR and lower pH value exhibited the intense effect on ICT release and about 77.34% of ICT was released after three times of NIR irradiation in pH4.5. The acidic microenvironment is one of the characteristics of the bone defect microenvironments, which can lead to the inhibition of osteoblast activity, the increase of osteoclast activity, and the inhibition of bone formation [39, 40]. The release of PO_4_^3−^ from the degradation of BP_ICT_ gradually increased with time, indicating that BP_ICT_ has good degradability (Additional file 1: Fig. S13). During the observing period, the release of PO_4_^3−^ in the BP_ICT_+NIR group remains consistently higher compared to the BP_ICT_ group. This indicated that NIR irradiation can enhance the degradation of BP_ICT_, promoting the release of PO_4_^3−^. Thus, ICT and PO_4_^3−^ will be rapidly released from BP-based nanomaterials in the bone defects microenvironment upon NIR irradiation, which would be advantageous to the regeneration of bone defect. Accordingly, the Ca element recruitment was examined on the surface of BP by TEM elemental mapping analysis. As shown in Fig. 2E and Additional file 1: Fig. S14, Ca element could be detected after the BP were incubated with SBF for 7 days. However, upon NIR irradiation, more Ca elements were recruited on the surface of BP and could reach up to 6.23% indicating that NIR exposure could accelerate the degradation of BP facilitating the recruitment of Ca element for bone repair. The results were confirmed by XRD demonstrating that the diffraction peaks of Ca_3_(PO_4_)_2_ (2θ = 20.074, 27.288, 31.507, 45.157) could be observed in the BP_ICT_+NIR group (Fig. 2F). In contrast, only the diffraction peaks of BP (2θ = 16.813, 26.451, 34.067, 52.176, 55.907) were observed in the control group (BP_ICT_ 0d). Similarly, in the room-temperature group (BP_ICT_ 7d), only the crystalline phases of BP changed (2θ = 16.736, 26.295, 33.921, 51.943, 55.596). These above results indicated that M@BP_ICT_ can absorb inflammatory factors and simultaneously provide bone repair factor and material for bone repair, achieving a one-step method for promoting osteogenesis. In addition, the microenvironment of bone defects is conducive to the degradation of BP_ICT_, which could facilitate the release of ICT and the formation of Ca_3_(PO_4_)_2_. Moreover, PTT derived from NIR irradiation could expedite this procedure and facilitate mineralization.

### Co-culture study of nanomaterials and BMSCs

**Fig. 3:**
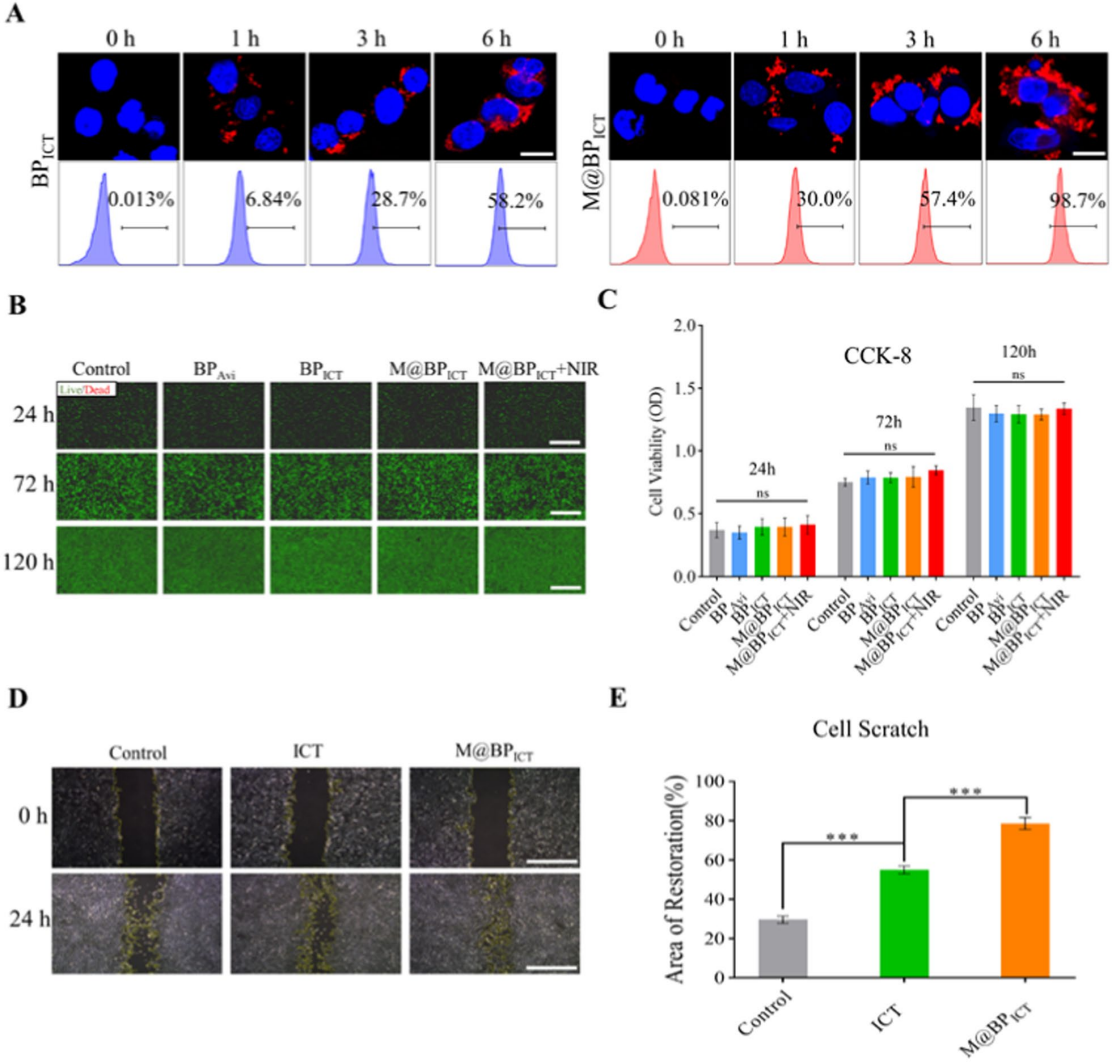
Co-culture Study of M@BP_ICT_ with BMSCs. **A** Fluorescence images of BMSCs treated with Cy5.5-modified BP_ICT_ and Cy5.5-modified M@BP_ICT_ for different times. (blue: DAPI, red: CY5.5, scale bars: 50 μm). **B** Live/Dead and **C** CCK-8 analysis of BMSCs cultured with various materials for 24, 72, and 120 h. (Green: live cells, Red: dead cells). NIR irradiation: 808 nm, 1.0 W cm^−2^, 10 min per 24 h. Scale bars: 200 μm. **D** Cell scratch assay and **E** quantitative analysis of migration area after BMSCs co-cultured with ICT or M@BP_ICT_ for 24 h. Scale bars: 200 μm. [BP_Avi_: 565.4 μg L^−1^, BP_ICT_: 1486.4 μg L^−1^, M@BP_ICT_: at the same BP_ICT_ concentration of 1486.4 μg L^−1^, calculated by 1 μM ICT. All statistical data are represented as mean ± SD (n = 6; ns *P* > 0.05; ***P < 0.001)]

Since M@BP_ICT_ might promote the bone regeneration, the endocytosis and biocompatibility were firstly evaluated on BMSCs, which could differentiate into osteoblast for the bone formation [41]. It was interesting to notice that the introduction of MMs could increase the endocytosis for BP_ICT_ indicated by the stronger fluorescence that nearly all of BMSCs endocytosed the Cy5.5-modified M@BP_ICT_ after 6 h of incubation. (Fig. 3A). Our previous research indicated that the drug-loaded BP encapsulated within MMs were almost entirely engulfed by cells. Therefore, when M@BP_ICT_ reaches the site of bone defects area, M@BP_ICT_ maybe uptake by cells via caveolae-dependent endocytosis [36]. Furthermore, the M@BP_ICT_ exhibited a prominent biocompatibility that the BMSCs were maintained alive indicated by the green fluorescence after being incubated with M@BP_ICT_ for 120 h (Fig. 3B, C). The NIR irradiation also did not show cytotoxicity to the BMSCs, which might be attributed to the moderated photothermal effect and the temperature was maintained below 42 °C. ICT could promote the migration of BMSCs via the MAPK signaling pathway to facilitate osteogenic differentiation at bone defect area [42, 43]. Therefore, whether the biotin-modification or loaded on BP would influence the activity of ICT on BMSCs were examined by the cell scratch assay. As shown in Fig. 3D, E, free ICT and M@BP_ICT_ could significantly promote the migration of BMSCs compared with PBS group. However, the M@BP_ICT_ induce more migration than the non-modified ICT, which might be contributed from the enhanced cellular uptake of the nanoparticles. The studies demonstrated that MMs-encapsulated BP_ICT_ with good biocompatibility could promote endocytosis and migration for BMSCs, thereby initiating and accelerating the process of bone regeneration.

### In vitro osteogenesis under inflammatory microenvironment

**Fig. 4:**
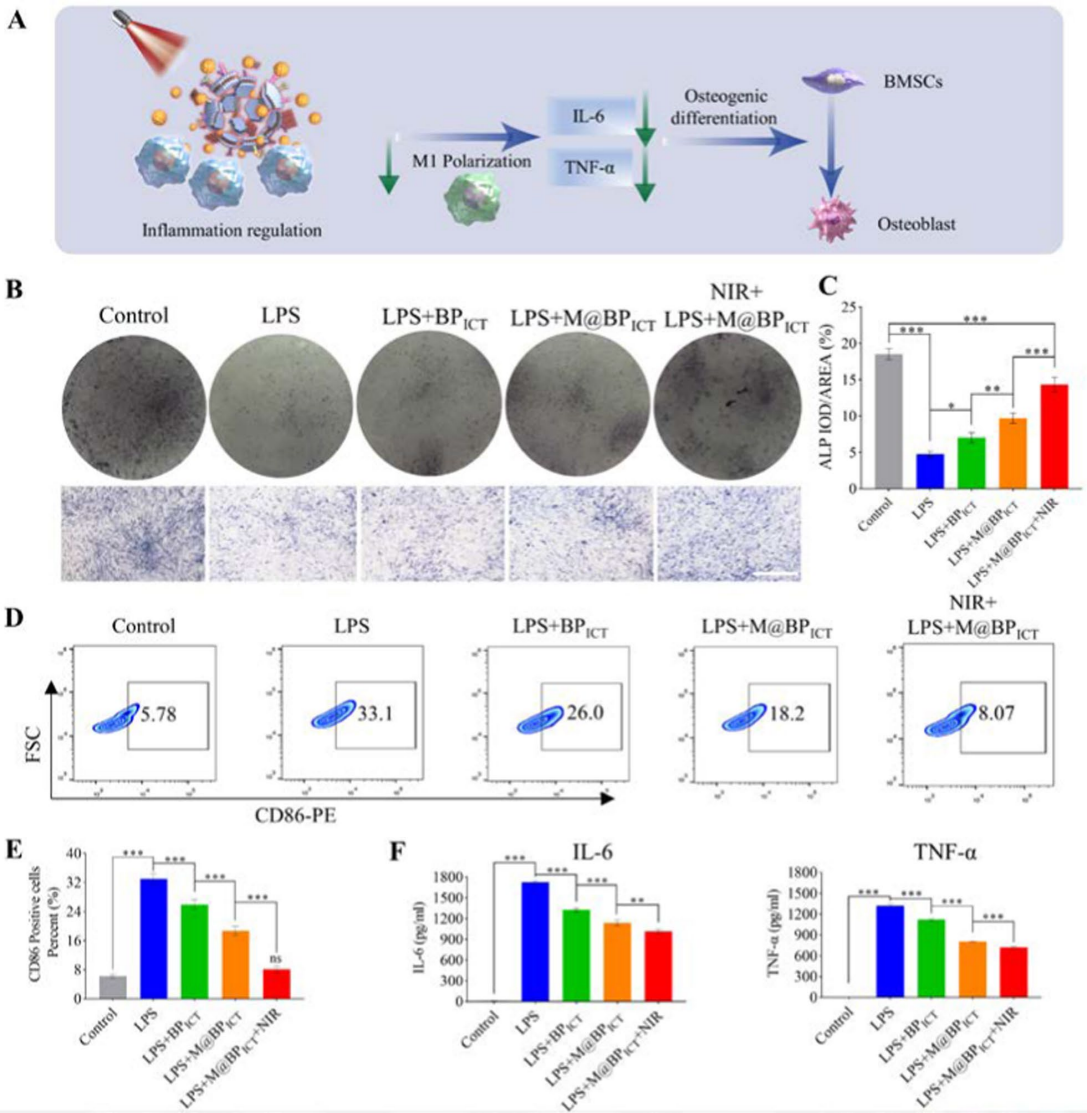
Osteogenic differentiation of BMSCs in inflammatory microenvironment induced by macrophage-conditioned medium and secretion of inflammatory factors by Raw 264.7 under different culture conditions. **A** Schematic diagram of inflammatory regulation of M@BP_ICT_. **B** ALP staining and **C** quantitative analysis of BMSCs cultured in conditioned medium for 14 days. The osteogenic induction medium under inflammatory microenvironment for the control group was formulated by combining Raw264.7 cell culture medium (without additional intervention) with regular osteogenic induction medium at a 1:2 ratio by volume. Scale bar: 200 μm. [LPS: 100 ng mL^−1^, BP_Avi_: 1118.4 μg L^−1^, BP_ICT_: 1486.8 μg L^−1^, M@BP_ICT_: at the same BP_ICT_ concentration of 1486.8 μg L^−1^, calculated by 1 μM ICT. **D** Representative dot plots of flow cytometry results. The LPS-incubated Raw 264.7 cells were treated with different formulations of BP for 48 h followed by staining with PE-labeled CD86. **E** The percentages of CD86-positive cells in different groups. **F** ELISA assay for IL-6 and TNF-α in in the supernatant of RAW264.7 cells with different treatments. Statistical data are represented as mean ± SD (n = 6; ns *P* > 0.05; *P < 0.05; **P < 0.01; ***P < 0.001)]

The acute phase of bone injury is usually accompanied by a large accumulation of pro-inflammatory factors, and pro-inflammatory factors (TNF-α, IL-6) could enhance osteoclast activity and impair bone regeneration. Therefore, regulation of inflammatory factor levels is a crucial method for promoting early-stage bone regeneration. To this aim, we evaluated whether M@BP_ICT_ could promote osteogenesis in the inflammatory microenvironment by simulating in vitro the early inflammatory microenvironment following bone defects in vivo (Fig. 4A) [34]. As shown in Fig. 4B, C, the ALP staining demonstrated that the osteogenic differentiation of BMSCs was significantly decreased by the treatment of LPS. In contrast, BP_ICT_ treatments could reverse LPS-induced reduction of osteogenic differentiation. Furthermore, M@BP_ICT_ exhibited stronger activity on the osteogenic differentiation indicated by the ALP staining, which could be enhanced by the NIR irradiation. The combined effect on osteogenic differentiation might be contributed the inflammatory factor absorption, anti-inflammatory activity, and enhanced mineralization. Accordingly, the inflammatory inhibition mediated by M@BP_ICT_ was examined by measuring the polarization of M0 macrophages. As shown in Fig. 4D, E, BP_ICT_ treatments could significantly decrease the proportion of LPS-stimulated M1 macrophages (CD86-positive). The anti-inflammatory activity could be originated from the ICT-mediated the regulation of inflammatory cytokines and phosphorylation of p38 and JNK [44, 45]. Consistent with previous results, MMs-encapsulated BP_ICT_ could further reduce the ratio of M1 macrophages, which could be significantly strengthened upon NIR exposure. The anti-inflammatory activity of M@BP_ICT_ was confirmed by determining the cytokines secreted from LPS-incubated macrophages. The combination of M@BP_ICT_ and NIR exposure exhibited the strongest inhibition on the expression of inflammatory factors including IL-6 and TNF-α (Fig. 4F). The results indicated that M@BP_ICT_ could inhibit the polarization of M1 macrophages and expression of inflammatory factors, which could enhance osteoclast activity and inhibit osteoblast activity, thereby inhibiting bone repair [46]. Consequently, the application of M@BP_ICT_ upon NIR irradiation might take full advantage of the inflammatory microenvironment to enable targeted delivery of drugs in the bone defects area. Subsequently, M@BP_ICT_ could improve the inhibition of osteogenic differentiation by inflammation through modulation of microenvironmental inflammatory factors.

### In vitro osteogenesis under inflammatory-free microenvironment

**Fig. 5:**
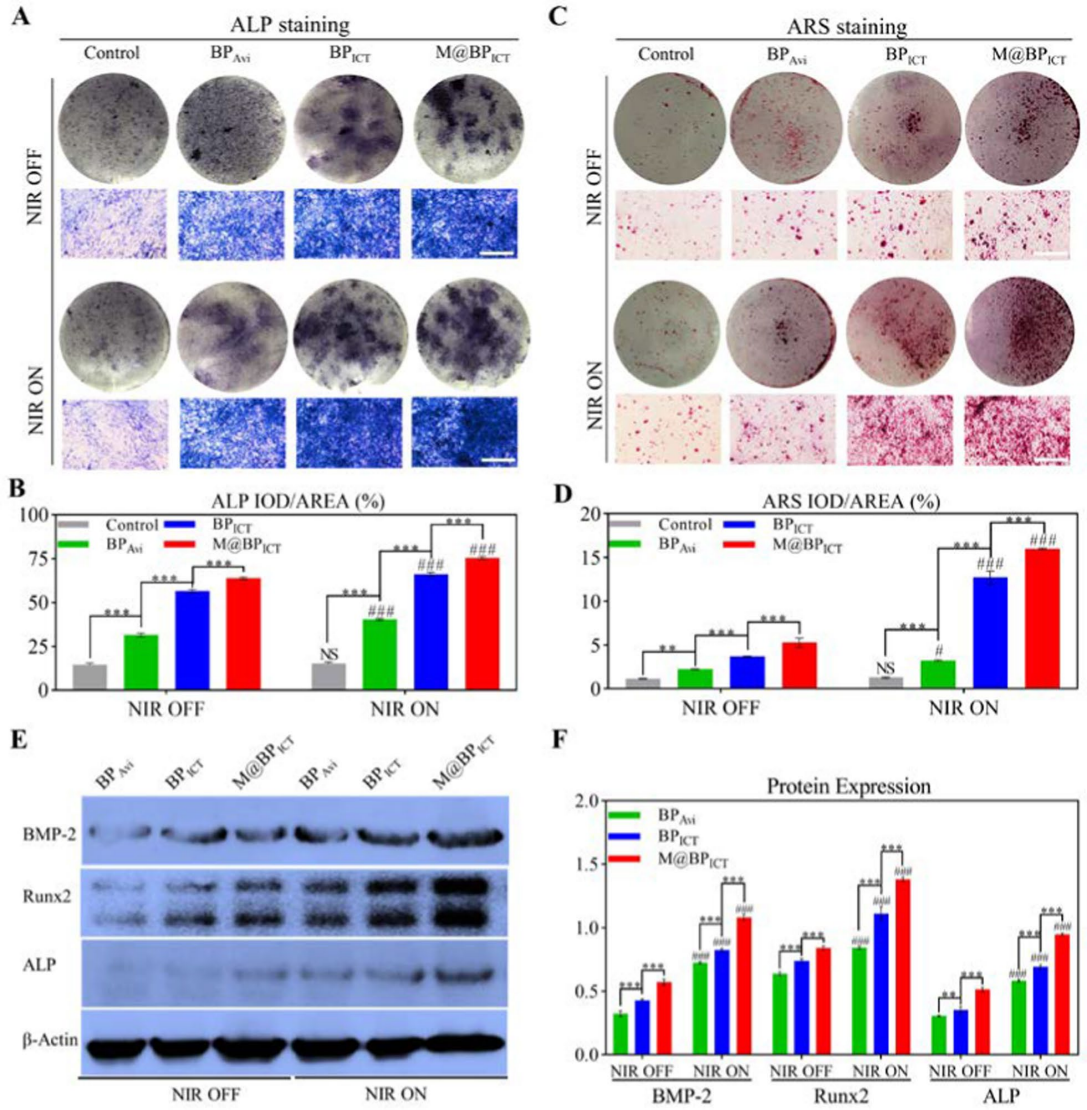
The in vitro osteogenic activities of M@BP_ICT_ on BMSCs*.* **A** The ALP staining images of BMSCs co-cultured with different formulations of BP with or without NIR irradiation (NIR ON or OFF) for 14 days. NIR irradiation: 808 nm, 1.0 W cm^−2^, 10 min per day. Scale bars: 200 μm. **B** Quantitative analysis of the ALP staining. **C** The ARS staining images of BMSCs co-cultured with different formulations of BP with or without NIR irradiation (NIR ON or OFF) for 21 days. NIR irradiation: 808 nm, 1.0 W cm^−2^, 10 min per day. Scale bars: 200 μm. **D** Quantitative analysis of ARS staining. **E** Western blot analysis and summarized quantitative data (**F**) for BMP-2, Runx2, and ALP of BMSCs co-cultured with different formulations of BP with or without NIR irradiation (NIR ON or OFF) for 7 d. NIR irradiation: 808 nm, 1.0 W cm^−2^, 10 min per day. [BP_Avi_: 565.4 μg L^−1^, BP_ICT_: 1486.4 μg L^−1^, M@BP_ICT_: at the same BP_ICT_ concentration of 1486.4 μg L^−1^, calculated by 1 μM ICT. All statistical data are represented as mean ± SD (n = 6; **P < 0.01; ***P < 0.001 compared between different groups in NIR ON or NIR OFF. NS *P* > 0.05; ^#^*P* < 0.05; ^###^*P* < 0.001 compared between NIR ON and NIR OFF in the same group)]

As bone repair progresses, the inflammatory microenvironment tends toward homeostasis. Therefore, the combined effect of M@BP_ICT_ and NIR on in vitro osteogenesis differentiation of BMSCs were examined under Inflammatory-free Microenvironment. The activity of ALP, a typical marker for the early stage of osteoblast differentiation was stained to analyze the activity of M@BP_ICT_. As shown in Fig. 5A, B, M@BP_ICT_ treatments are more conducive to the osteogenic differentiation of BMSCs compared with PBS or avidin-connected BP after 14 days of osteogenesis induction. More importantly, the application of NIR irradiation could significantly improve the osteogenic differentiation of BMSCs treated with BP-based nanomaterials, indicating that moderate PTT is an effective osteogenesis strategy. The degradation of BP was advantageous during the process of bone repair. The ARS staining was performed to evaluate the degree of mineralization in BMSCs with different treatments. As shown in Fig. 5C, D, after 21 days of osteogenic induction, relatively few and small mineralized nodules were detected in the control group, whereas abundant mineralized nodules were observed in the BP-treated groups. Except for the control group, more mineralized nodules could be detected in the NIR-irradiated groups than the group without NIR exposure. The reinforced effect of mineralization might be a result of PTT-mediated osteogenesis promotion and the formation of Ca_3_(PO_4_)_2_ from BP degradation. Furthermore, the results were confirmed by the examining the expression of osteogenesis-related proteins demonstrating that M@BP_ICT_ exhibited the strongest activity on the induction of BMP-2, Runx2, and ALP, which could be significantly increased by NIR irradiation (Fig. 5E, F). Overall, the M@BP_ICT_+NIR group exhibited greater osteogenic potential in vitro than other groups.

### RNA sequencing (RNA-seq) analysis of the BP_ICT_ for osteogenic differentiation

**Fig. 6:**
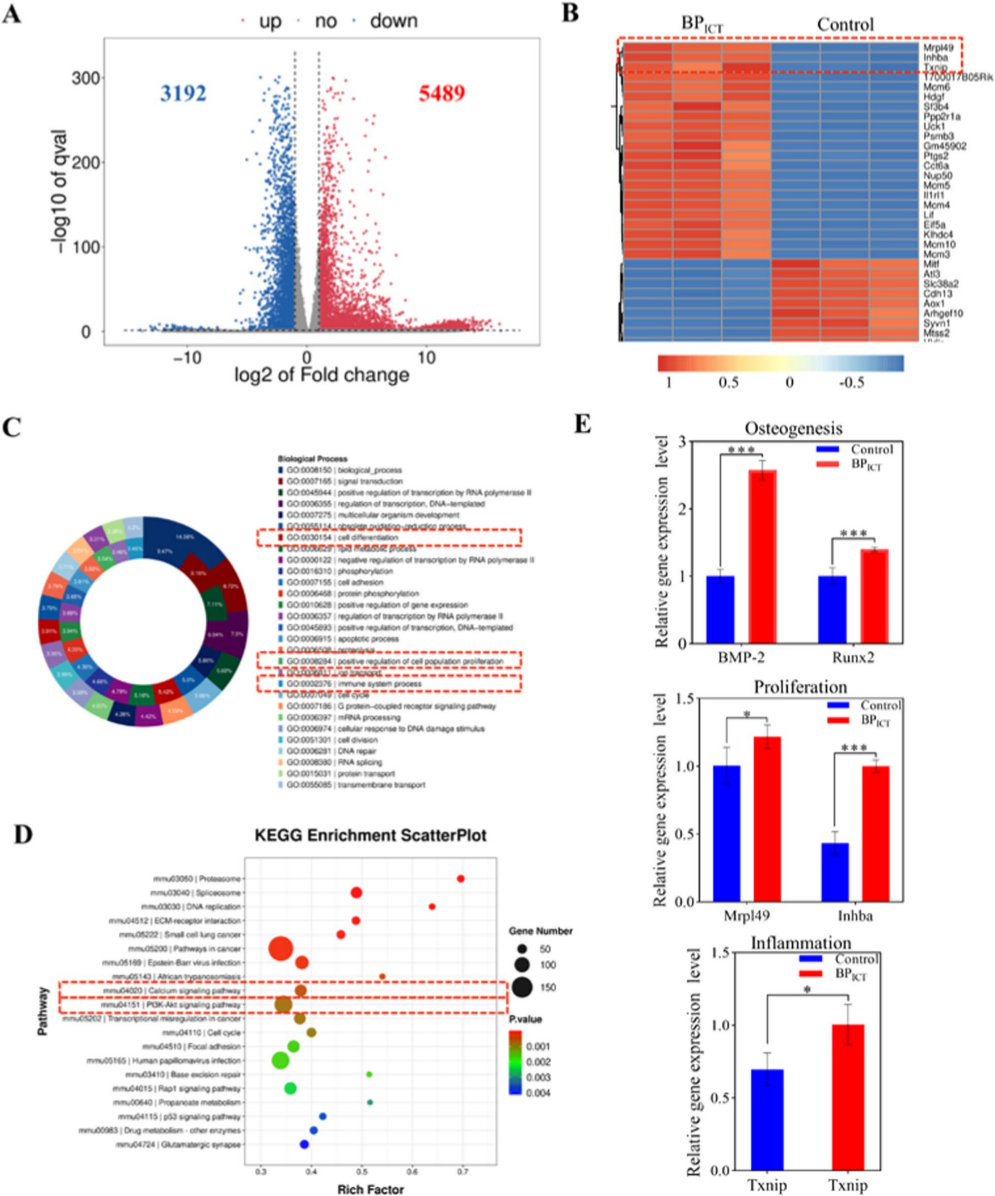
RNA-seq test and qPCR analysis. **A** Volcano plot of DEGs. Blue and red represented down-regulated and up-regulated genes, respectively. **B** Hierarchical clustering analysis of DEGs. **C** GO functional analysis of DEGs. **D** KEGG enrichment analysis of DEGs. The bubble size reflected the gene counts enriched in each term, and color represented the p value. **E** The relative gene expression levels of BMP-2, Runx2, Mrpl49, Inhba and Txnip. Data were presented as the mean ± SD, *P < 0.05, **P < 0.01, ***P < 0.001 (n = 3)

To further investigate the molecular mechanisms of BP_ICT_ on the osteogenic differentiation and mineralization of BMSCs, we performed RNA-seq analysis on BMSCs cultured with BP_ICT_ intervention. Figure 6A presented the volcano plot of differentially expressed genes (DEGs), where gray indicates genes with non-significant differences, red and blue represent genes with significantly altered expression. Among the DEGs, 5489 genes were upregulated (red), and 3192 genes were downregulated (blue). Based on the top 100 DEGs shown in the heatmap (Fig. 6B), when compared to the control group, significant upregulation was observed in mitochondrial ribosomal protein L49 (Mrpl49), inhibin subunit beta A gene (Inhba), and thioredoxin-interacting protein (Txnip). Figure 6C presented the results of the Gene Ontology (GO) analysis, indicating that BP_ICT_ can exert osteogenic effects through cell differentiation, positive regulation of cell population proliferation, and immune system processes. Additionally, Kyoto Encyclopedia of Genes and Genomes (KEGG) pathway analysis was conducted (Fig. 6D). The enrichment level was shown on the horizontal axis, with larger bubbles indicating more DEGs. The calcium signaling pathway and PI3K-AKT signaling pathway showed high enrichment, suggesting that BP_ICT_ may accelerate osteogenesis through these pathways. The PI3K-AKT signaling pathway may play a role in regulating cell proliferation, bone metabolism, and macrophage polarization. Furthermore, the upregulation of the calcium signaling pathway further supports the involvement of phosphate ions and calcium ions released by BP_ICT_ in promoting bone defect repair.

Subsequently, real-time quantitative polymerase chain reaction (qPCR) analysis was performed to detect and validate the transcriptionally related genes (Mrpl49, Inhba and Txnip in Fig. 6E) identified from the transcriptomic analysis, as well as the osteogenic-related genes (BMP-2 and Runx2 in Fig. 6E). Mrpl49 is one of the mitochondrial ribosomal proteins involved in the synthesis and function of mitochondrial proteins. Its interaction with the PI3K-AKT pathway and bone metabolism has not been confirmed yet. However, mitochondria serve as the energy powerhouse within cells and are closely associated with cellular metabolism. Therefore, we speculate that Mrpl49 primarily exerts its osteogenic effects through the regulation of mitochondrial function, cell proliferation, and survival. The PI3K-AKT pathway may indirectly impact cell proliferation and survival by influencing Mrpl49. Inhba is an important growth factor belonging to the TGF-β superfamily. It participates in the regulation of cell proliferation, differentiation, and other biological processes. Inhba also plays a role in modulating molecular signaling pathways associated with bone cell proliferation, differentiation, and bone formation. Therefore, the increased expression of Inhba during osteogenesis may contribute to the promotion and protection of bone formation. The PI3K-AKT pathway exhibits significant interaction with Inhba, involving the regulation of Inhba expression, signaling pathway modulation, cell proliferation, and survival [47, 48]. Txnip is a crucial regulatory protein involved in multiple biological processes, including cellular oxidative stress, glucose metabolism, apoptosis, and inflammation. Some studies have indicated its involvement in the regulation of osteoblasts, particularly in relation to cell proliferation, differentiation, and bone matrix formation. Additionally, Txnip is also associated with the PI3K-AKT pathway, specifically in the context of oxidative stress and apoptosis [49].

BMP-2 and Runx2 can both be activated through the PI3K-AKT signaling pathway [50, 51]. BMP-2 is a potent osteogenic inducer, and the PI3K-AKT pathway can promote the transcription and expression of BMP-2, thereby influencing osteogenic differentiation and bone formation during the osteogenesis process. Runx2 is a transcription factor that is essential for osteoblast differentiation, and the PI3K-AKT pathway can impact the activity and regulation of Runx2 through various mechanisms. In addition to the qPCR results (Fig. 6E), the elevation of BMP-2 and Runx2 proteins in cellular experiments (Fig. 5E) aligns with the sequencing results. In conclusion, BP_ICT_ can promote cell proliferation, osteogenic differentiation, and osteogenic behavior of bone cells through the PI3K-AKT signaling pathway, while also exerting immunomodulatory effects.

### In vivo bone defects targeting ability and photothermal effects

**Fig. 7:**
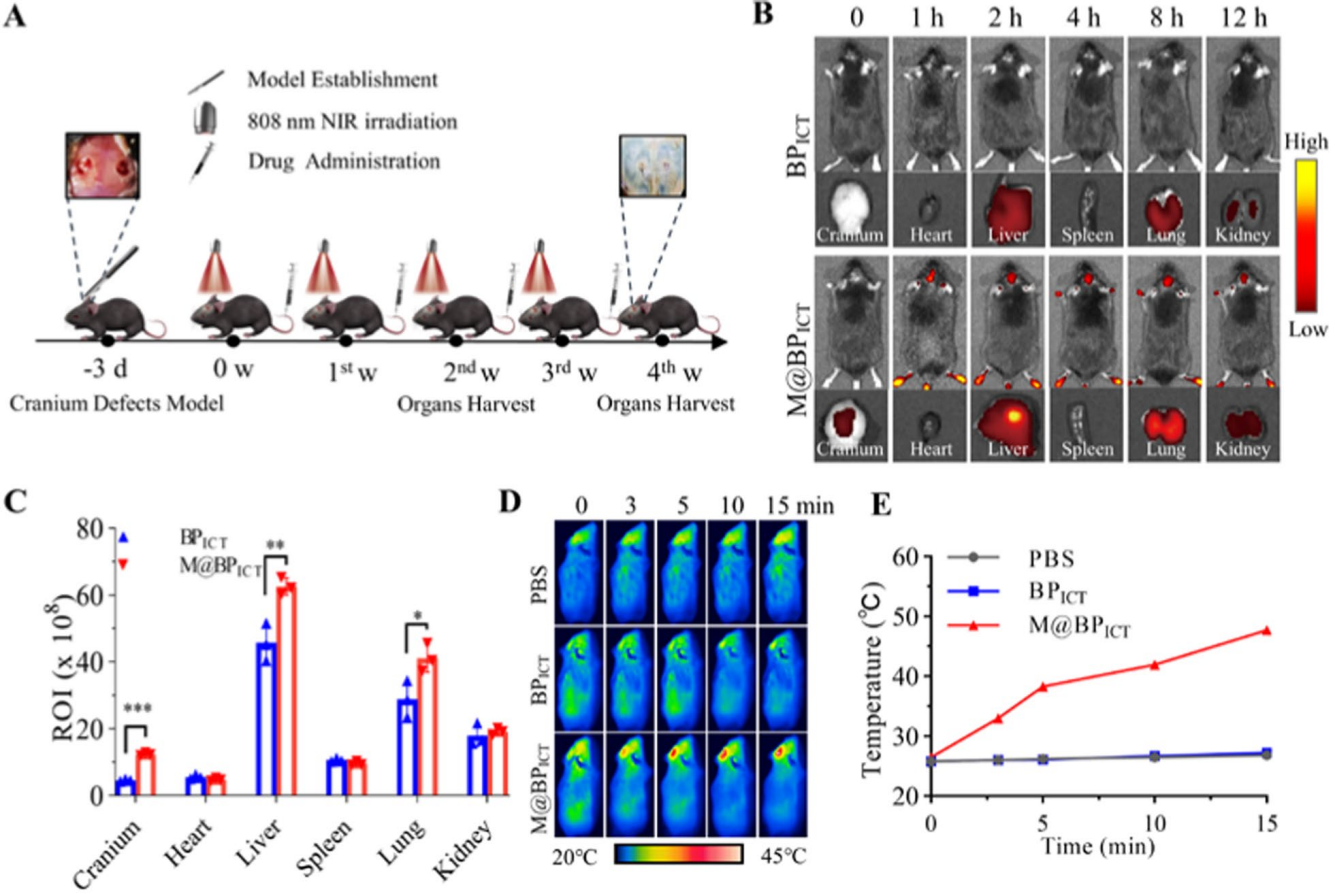
In vivo evaluation of bone-defect-targeting ability and photothermal performance. **A** Schematic illustration of the M@BP_ICT_-based PTT to accelerate bone regeneration. **B** In vivo fluorescence images for the biodistribution and dissociated major organs from mice injected with Cy5.5-modified BP_ICT_ with or without MMs encapsulation. **C** Quantitative analysis of the fluorescence distributed in the major organs. **D** NIR image and temperature curve (**E**) for the cranium defects with PBS, BP_ICT_ and M@BP_ICT_ upon different time of irradiation. [NIR irradiation: 808 nm, 1.0 W cm^−2^,10 min each time. Intravenous injection: PBS: 100 μL, BP_ICT_: 32.3 mg kg^−1^ and M@BP_ICT_: 32.3 mg kg^−1^ body weight, calculated by 8 mg kg^−1^ ICT, 100 μL each time. All statistical data are represented as mean ± SD (n = 3, ***P < 0.001)]

The MMs could enable BP_ICT_ to target the inflammatory microenvironment and release the cargos in responsive acid and NIR exposure. Therefore, the in vivo bone-defect-target ability of M@BP_ICT_ was investigated since the bone defect area was characterized with inflammatory microenvironments. A non-critical defect with a size of 3 mm was established on the murine cranium to simulate the region of post-traumatic bone defects (Fig. 7A) [35]. The mouse was performed with intravenous administration of Cy5.5-modified BP_ICT_ and M@BP_ICT_ and then monitored with In Vivo Imaging Systems (IVIS) at different time intervals. As shown in Fig. 7B, C, the fluorescence around the cranium could be observed after 1 h of injection with the M@BP_ICT_, whereas no fluorescence signal was detected in the cranium for the non-encapsulated BP_ICT_. After 12 h of injection, the major organs including brain tissues were separated for ex vivo fluorescence analysis. The results demonstrated that the M@BP_ICT_ could be detected at the brain tissues, whereas the brain with the treatments of BP_ICT_ was absence of fluorescence indicating that the introduction of MMs could help BP_ICT_ to target the bone defects area. Besides, Strong fluorescent signals could be observed in the liver, lung and limbs of the mouse in the M@BP_ICT_ group, which may be related to the distribution characteristics of macrophages in the body [52–55]. More importantly, M@BP_ICT_ was able to mediate the photothermal effect as indicated by the gradually increasing temperature at the cranium defects area upon NIR irradiation monitored with an infrared thermal camera (Fig. 7D). M@BP_ICT_ exhibited a moderate photothermal effect and could reach up to 41.9 °C within 10 min of NIR irradiation (Fig. 7E). Consequently, the temperature of the bone defects area was maintained within 40 to 42 °C in this study since high temperature might cause cell damage and be disadvantageous to the bone regeneration [56]. Furthermore, after 4 weeks of continuous treatment with M@BP_ICT_+NIR, the photothermal effect could still be detected indicating M@BP_ICT_ possessed the ability to durably target bone defects (Additional file 1: Fig. S15). In conclusion, M@BP_ICT_ exhibited superior ability to target bone defects area and photothermal effect in vivo.

### Radiological evaluation of bone regeneration in vivo

**Fig. 8:**
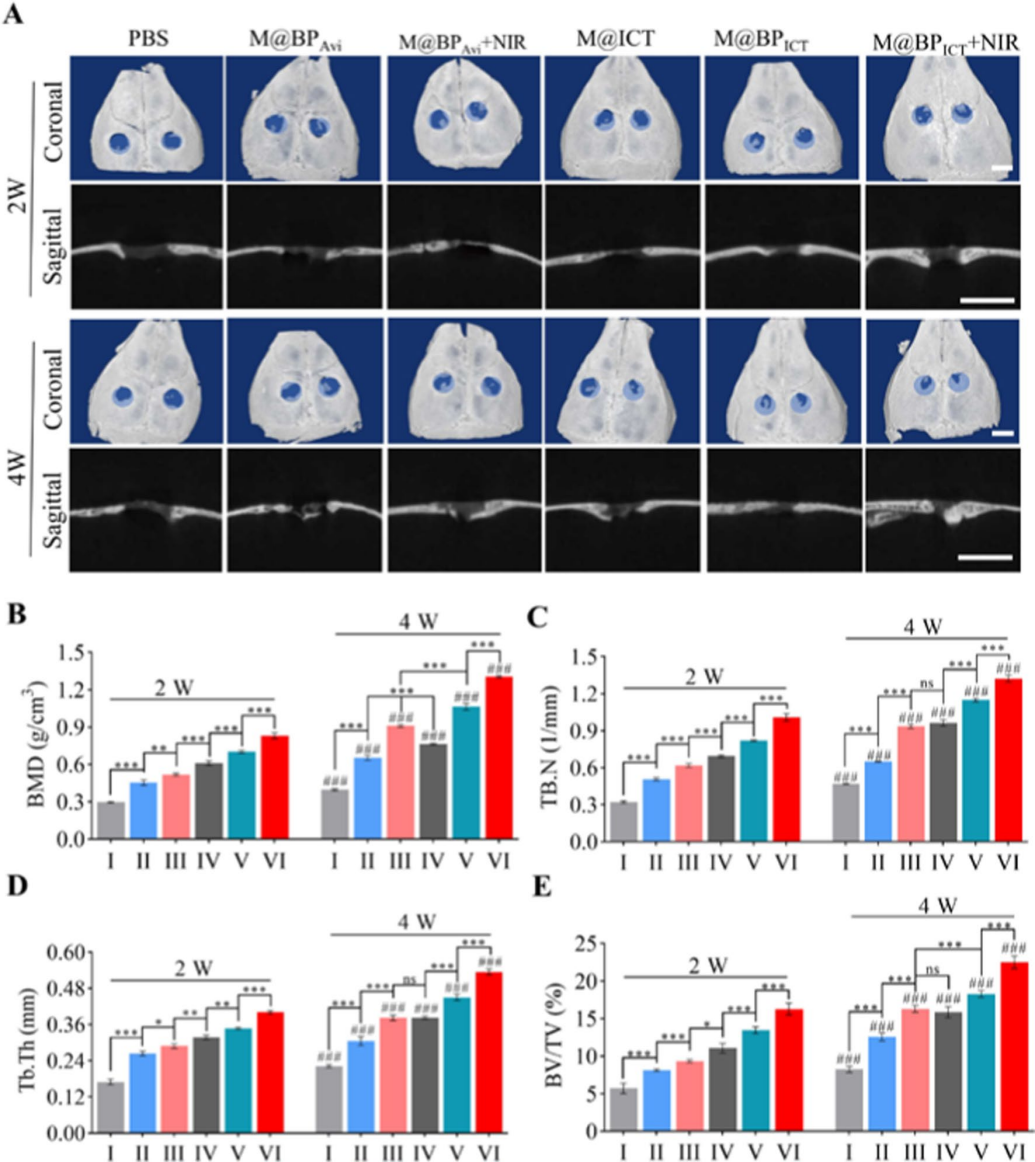
Micro-CT analysis of bone indexes for mice with cranium defects after different treatments. **A** Representative micro-CT reconstruction images in coronal and sagittal view in 2nd and 4th week for different groups [PBS (I), M@BP_Avi_ (II), M@BP_Avi_+NIR (I, II), M@ICT (IV), M@BP_ICT_ (V), M@BP_ICT_+NIR (VI)]. The initial boundary of cranium defects was marked by light blue area. Scale bars: 3 mm. Quantitative analysis of bone regeneration effect after different treatments, including BMD (**B**), TB.N (**C**), Tb. Th (**D**) and BV/TV (**E**). [NIR irradiation: 808 nm, 1.0 W cm^−2^, 10 min, under anesthesia 6 h after administration. Dosages of injection: PBS: 100 μL, M@BP_Avi_: 24.3 mg kg^−1^, M@ICT: 8 mg kg^−1^ and M@BP_ICT_: 32.3 mg kg^−1^ body weight, calculated by 8 mg kg^−1^ ICT, 100 μL each time, once every week, i. v. All statistical data are represented as mean ± SD (n = 3. ns *P* > 0.05; *P < 0.05; **P < 0.01; ***P < 0.001 compared between different groups in 2W or 4W. ^###^*P* < 0.001 compared between 2 and 4W in the same group)]

The M@BP_ICT_ could efficiently target the bone defect tissues and exhibit photothermal performance. Accordingly, the combination of M@BP_ICT_ and NIR irradiation on bone repair was evaluated on the mouse with non-critical size cranium defects (Fig. 8A). The model mice were randomly divided into 6 groups (n = 6): PBS group, M@BP_Avi_, M@BP_Avi_+NIR group, M@ICT group, M@BP_ICT_ group, M@BP_ICT_+NIR group. Each group of mice were performed with one intravenous injection of different formulations (100 μL) per week. At the 2nd and 4th weeks postoperatively, the micro-CT scanning and three-dimensional (3D) reconstructions of the cranium defects area were conducted to evaluate the bone repair. As shown in Fig. 8A, the repairing progress of bone defects in the 4th week was superior compared with the 2nd week. At the end of the 2nd week, M@BP_Avi_, M@BP_ICT_, M@ICT, M@BP_ICT_, and M@BP_ICT_+NIR all exhibited significant activity on the bone regeneration (light blue circle area) compared with the PBS group, indicating that the self-healing of bone defect in mice was seriously inadequate. In contrast, the combination of M@BP_ICT_ and NIR exposure exhibited the strongest activity on the bone regeneration as evidenced by the greatest quantity of new bone tissue and the smallest remaining bone defects area. The quantitative analysis for the bone parameters positively correlated with the quality of bone repair demonstrated that the M@BP_ICT_+NIR group exhibited the highest callus mineral bone density (BMD), trabecular number (TB. N), trabecular thickness (Tb. Th) and bone volume per total volume (BV/TV) at both of the time intervals (Fig. 8B–E). At the 4th week, the M@BP_Avi_ group showed higher BMD compared with the M@ICT group, indicating that the increased bone density might be contributed from the bone repair material of M@BP_Avi_. Furthermore, NIR irradiation further improved the bone repair effect. Overall, multifunctional nanomaterials (M@BP_ICT_) had a significantly better bone regeneration effect than nanomaterials delivering only bone repair material (M@BP_Avi_) or bone growth factor (M@ICT). Additionally, appropriate PTT could enhance bone regeneration.

### Histological evaluation of bone regeneration in vivo

**Fig. 9:**
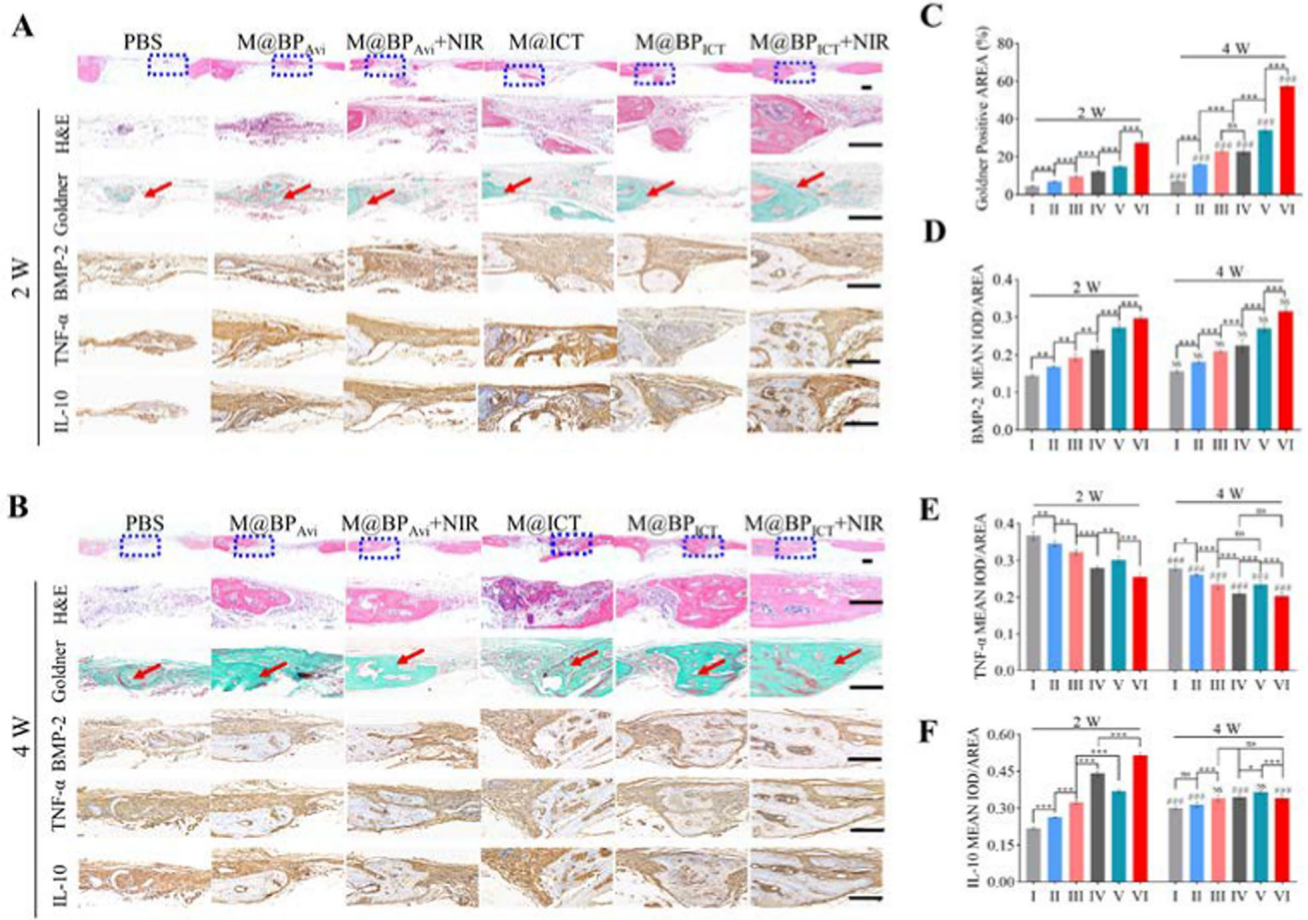
Bone tissue analysis for the cranium defects to bone regeneration. H&E staining, Goldner staining, immunohistochemistry staining of BMP-2, TNF-α, and IL-10 staining at 2nd week (**A**) and 4th week (**B**). The staining images are higher magnifications of the areas within the blue boxes. Red arrow showed new bone. Scale bars are 1000 μm in original H&E staining, 100 μm under high magnification images. Positive areas of Goldner (**C**), BMP-2 (**D**), TNF-α (**E**) and IL-10 (**F**) staining were measured by Image J software. (NIR irradiation: 808 nm, 1.0 W cm^−2^, 10 min, under anesthesia 6 h after administration. Dosages of injection: PBS: 100 μL, M@BP_Avi_: 24.3 mg kg^−1^, M@ICT: 8 mg kg^−1^ and M@BP_ICT_: 32.3 mg kg^−1^ body weight, calculated by 8 mg kg^−1^ ICT, 100 μL each time, once every week, i.v. All statistical data are represented as mean ± SD (n = 3. ns *P* > 0.05; *P < 0.05; **P < 0.01; ***P < 0.001 compared between different groups in 2W or 4W. NS *P* > 0.05; ^*#*^*P* < 0.05; ^##^*P* < 0.01; ^###^*P* < 0.001 compared between 2 and 4W in the same group)]

The M@BP_ICT_ could significantly promote the bone repair based on anti-inflammatory activity, which could be further increased by the NIR irradiation. Therefore, the histological staining was performed to confirm the bone-repaired effect and in vivo anti-inflammatory activity. The H&E and Goldner staining demonstrated that the bone defect treated with the combination of M@BP_ICT_ and NIR irradiation had been near completely repaired by new bone tissues on the 4th week (Fig. 9A–C). Furthermore, the immunohistochemical staining and western blot assay demonstrated the expression of BMP-2 was significantly increased in M@BP_ICT_+NIR group compared with other groups (Fig. 9D and Additional file 1: Fig. S16), which was consistent with bone regeneration. The in vivo anti-inflammatory activity was evaluated by the immunohistochemical staining of inflammatory TNF-α and IL-10. As shown in Fig. 9A, B, E and F, M@BP_ICT_+NIR treatments could significantly reduce the expression of pro-inflammatory factors of TNF-α and increase the expression of anti-inflammatory factors of IL-10. In addition, the biocompatibility of M@BP_ICT_ was evaluated by monitoring body weights of the mice with different treatments (Additional file 1: Fig. S17) and H&E staining of the major organs (heart, liver, spleen, lung and kidney) at the end of experiments (Additional file 1: Fig. S18). The results demonstrated the bodyweights in all the groups exhibited a gradual growth tendency without statistical difference and no pathological changes in the major organs. Overall, the combined application of M@BP_ICT_ and NIR irradiation exhibited the strongest activity for inflammatory inhibition and bone repair without significant systemic toxicity.

## Conclusion

In this study, a multifunctional bone defect area targeting nanocarrier delivery system (BTNDS) was engineered for active targeted bone repair treatment in bone defects area. The system was characterized with bone-defect-target capacity, inflammatory factors absorbing ability, increased mineralization, acid-and NIR-responsive drug release behaviors for the bone repair. The experimental results indicated that the nanomaterials have the appropriate sizes, and the concentrations used are within the safe range. Furthermore, the final degradation products of BP are non-toxic PO_4_^3−^. Thus, the nanomaterials could exhibit good biocompatibility. The ICT was taken as a modal drug loaded on the BP via the interaction of biotin and avidin, while BP could impart NIR responsiveness to BTNDS and provide PO_4_^3−^ as bone repair material. The combination of M@BP_ICT_ and NIR exposure could exhibit significant in vitro anti-inflammatory activity and elevated mineralization, thereby promoting bone repair. In a murine bone defect model, M@BP_ICT_ could efficiently target and accumulate at the bone defect tissues facilitating the drug release and photothermal performance. Upon the NIR irradiation, M@BP_ICT_ could significantly decrease the inflammatory reaction and accelerate the bone regeneration. The beneficial effects of M@BP_ICT_ mentioned above provide a promising solution for the clinical non-surgical effective delivery of bone repair factors and materials to treat bone defects. Its application prospects lie in the treatment of non-critical bone defects and in patient intolerant to surgical procedures for bone defects. Overall, the engineered M@BP_Avi_ could serve as a multifunctional drug delivery platform for inflammation intervention and provide a pivotal strategy for the management of bone defects.

### Supplementary Information

**Additional file 1: Figure S1.** CCK8 results of BMSCs cultured with different concentrations of ICT for 24 h. [All statistical data are represented as mean ± SD (n = 6; *P < 0.05)]. **Figure S2.** Results of ALP staining after 7 days of osteogenic induction with different concentrations of ICT, scale bar: 200 μm. [All statistical data are represented as mean ± SD (n = 6; ***P < 0.001)]. **Figure S3.** The synthetic route for biotin-connected ICT_._ **Figure S4.** The ^1^H NMR of biotin-connected ICT. Methylene hydrogen (–CH_2_–) was detected in the biotin unit at δ 0.87, 1.04 and 1.26 ppm and methylene hydrogen (-CH_3_) in the ICT unit was at δ 1.57–1.89 ppm. **Figure S5.** The synthetic route of BP_Avi._ **Figure S6.** XRD of BP, BP_Avi_ and BP_ICT_. **Figure S7.** The AFM image of BP. **Figure S8.** The SEM images of different formulations of BP. Scale bars: 100 nm. **Figure S9.** Quantitative results of elemental analysis of BP_Avi_ and BP_ICT_ (without SBF). **Figure S10.** The size distribution and morphology of BP (A) and BP_Avi_ (B) determined by DLS and TEM, scale bars: 100 nm. **Figure S11.** Zeta potential of BP, BP_Avi_, ICT_Bio_ and BP_ICT_. **Figure S12.** Photothermal conversion efficiency measurement. (A) Photothermal effect of the irradiation of the BP_ICT_ and M@BP_ICT_ (NIR: 808 nm, 2.0 W cm^−2^, BP: 0.1 mg, 800 μL), in which the irradiation lasted for 5 min, and then the laser was shut off. (B) Photothermal conversion efficiency (ƞ) of BP_ICT_ and M@BP_ICT_. Time constant for heat transfer from the system is determined by applying the linear time data from the cooling period (after 300 s) versus the negative natural logarithm of the driving force temperature, which is obtained from the cooling stage of (A). **Figure S13.** The cumulative release of P0_4_^3−^ from BP_ICT_ with or without NIR irradiation. (BP_ICT_, 1486.8 μg L^−1^, calculated by 1 μM ICT. NIR irradiation condition: 808 nm, 1.0 W cm^−2^, 10 min per day). **Figure S14.** Quantitative results of elemental analysis of BP_Avi_ and BP_ICT_ (with SBF). **Figure S15.** In vivo photothermal effect of M@BP_ICT_ was maintained for 4 weeks. **Figure S16.** Representative western blot images of BMP-2. **Figure S17.** The body weight of mice with different treatments. **Figure S18.** Representative H&E staining images of major organs including heart, liver, spleen, lung, and kidney collected from different groups. Scale bars: 200 μm. **Table S1.** The Primer sequences used in this study.

## Data Availability

The data that support the findings of this study are available from the corresponding author upon reasonable request.

## Abbreviations

BTNDS: Bone defect area targeting nanocarrier delivery system
PTT: Photothermal therapy
BNDS: Bone-targeting nanocarrier systems
MMs: Macrophage-derived membranes
2D: Two-dimensional
BMSCs: Bone marrow mesenchymal stem cells
NIR: Near infrared
IVIS: In vivo imaging systems
BMD: Bone mineral density
Tb. N: Trabecular number
Tb. Th: Trabecular thickness
BV/TV: Bone tissue volume/total tissue volume
FTIR: Fourier Transform Infrared Spectrometer
TGA: Thermogravimetric analysis
SEM: Scanning Electron Microscope
AFM: Atomic Force Microscope
TEM: Transmission Electron Microscope
EDS: Energy dispersive X-ray spectroscopy
DLS: Dynamic light scattering
XRD: X-ray diffraction
FCM: Flow cytometry
ALP: Alkaline phosphatase
BMP-2: Bone morphogenetic protein 2
Runx2: Runt-related transcription factor-2
ARS: Alizarin red S
