# Supplementary material for: Macrophage membrane (MMs) camouflaged near-infrared (NIR) responsive bone defect area targeting nanocarrier delivery system (BTNDS) for rapid repair: promoting osteogenesis via phototherapy and modulating immunity
Source: J Nanobiotechnology. 2024 Mar 1;22:87. doi: 10.1186/s12951-024-02351-5 (PMC10908146; doi:10.1186/s12951-024-02351-5)
Supplement: Supplementary file 1 — Additional file 1: Figure S1. CCK8 results of BMSCs cultured with different concentrations of ICT for 24 h. [All statistical data are represented as mean ± SD (n = 6; *P < 0.05)]. Figure S2. Results of ALP staining after 7 days of osteogenic induction with different concentrations of ICT, scale bar: 200 μm. [All statistical data are represented as mean ± SD (n = 6; ***P < 0.001)]. Figure S3. The synthetic route for biotin-connected ICT. Figure S4. The 1H NMR of biotin-connected ICT. Methylene hydrogen (–CH2–) was detected in the biotin unit at δ 0.87, 1.04 and 1.26 ppm and methylene hydrogen (-CH3) in the ICT unit was at δ 1.57–1.89 ppm. Figure S5. The synthetic route of BPAvi. Figure S6. XRD of BP, BPAvi and BPICT. Figure S7. The AFM image of BP. Figure S8. The SEM images of different formulations of BP. Scale bars: 100 nm. Figure S9. Quantitative results of elemental analysis of BPAvi and BPICT (without SBF). Figure S10. The size distribution and morphology of BP (A) and BPAvi (B) determined by DLS and TEM, scale bars: 100 nm. Figure S11. Zeta potential of BP, BPAvi, ICTBio and BPICT. Figure S12. Photothermal conversion efficiency measurement. (A) Photothermal effect of the irradiation of the BPICT and M@BPICT (NIR: 808 nm, 2.0 W cm−2, BP: 0.1 mg, 800 μL), in which the irradiation lasted for 5 min, and then the laser was shut off. (B) Photothermal conversion efficiency (ƞ) of BPICT and M@BPICT. Time constant for heat transfer from the system is determined by applying the linear time data from the cooling period (after 300 s) versus the negative natural logarithm of the driving force temperature, which is obtained from the cooling stage of (A). Figure S13. The cumulative release of P043− from BPICT with or without NIR irradiation. (BPICT, 1486.8 μg L−1, calculated by 1 μM ICT. NIR irradiation condition: 808 nm, 1.0 W cm−2, 10 min per day). Figure S14. Quantitative results of elemental analysis of BPAvi and BPICT (with SBF). Figure S15. In vivo photothermal effect o [file 12951_2024_2351_MOESM1_ESM.docx]

**Additional file 1**

**Macrophage Membrane (MMs) Camouflaged Near-infrared (NIR) Responsive Bone Defect Area Targeting Nanocarrier Delivery System (BTNDS) for Rapid Repair: Promoting Osteogenesis via Phototherapy and Modulating Immunity**

**
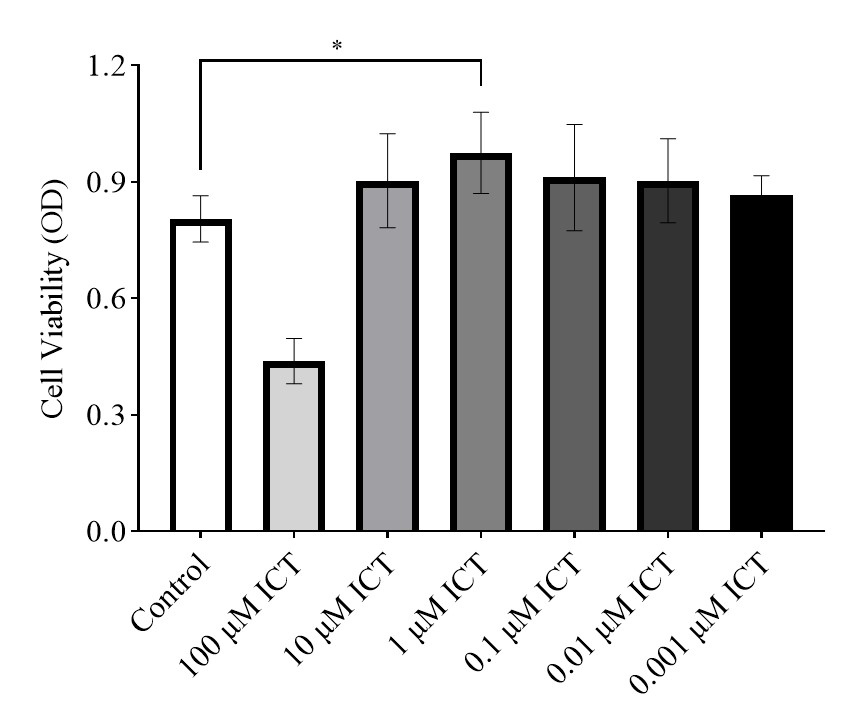
**

**Figure S1.** CCK8 results of BMSCs cultured with different concentrations of ICT for 24 hours. (All statistical data are represented as mean ± SD (n = 6; **P*<0.05)).

**
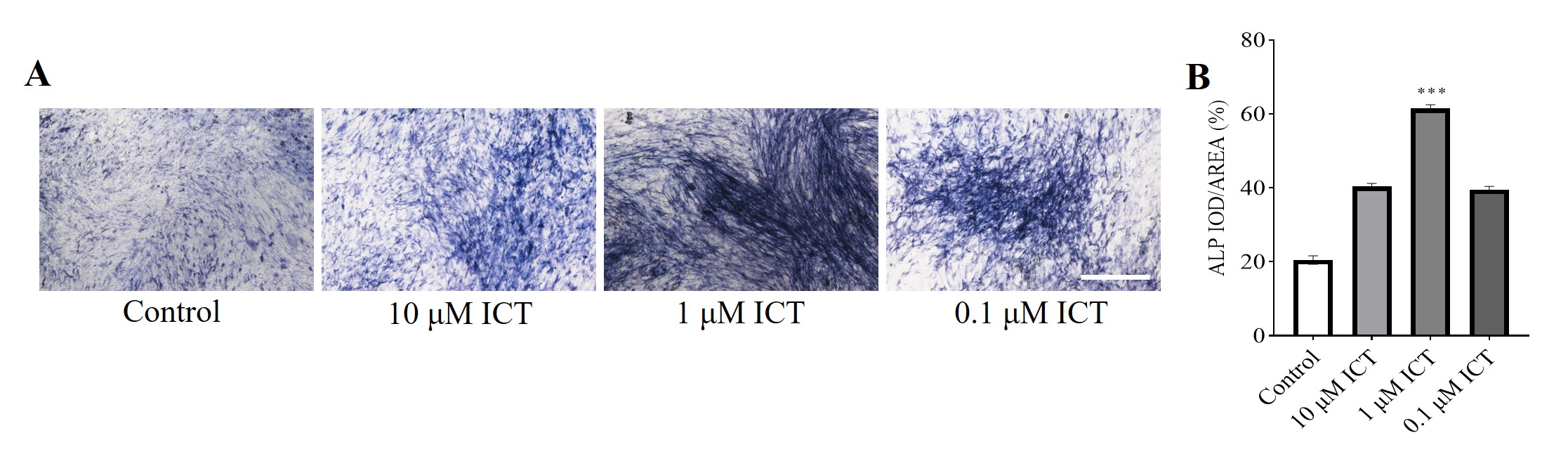
**

**Figure S2.** Results of ALP staining after 7 days of osteogenic induction with different concentrations of ICT, scale bar: 200 μm. (All statistical data are represented as mean ± SD (n = 6; ****P*<0.001)).

**Figure S3.** The synthetic route for biotin-connected ICT_._

**
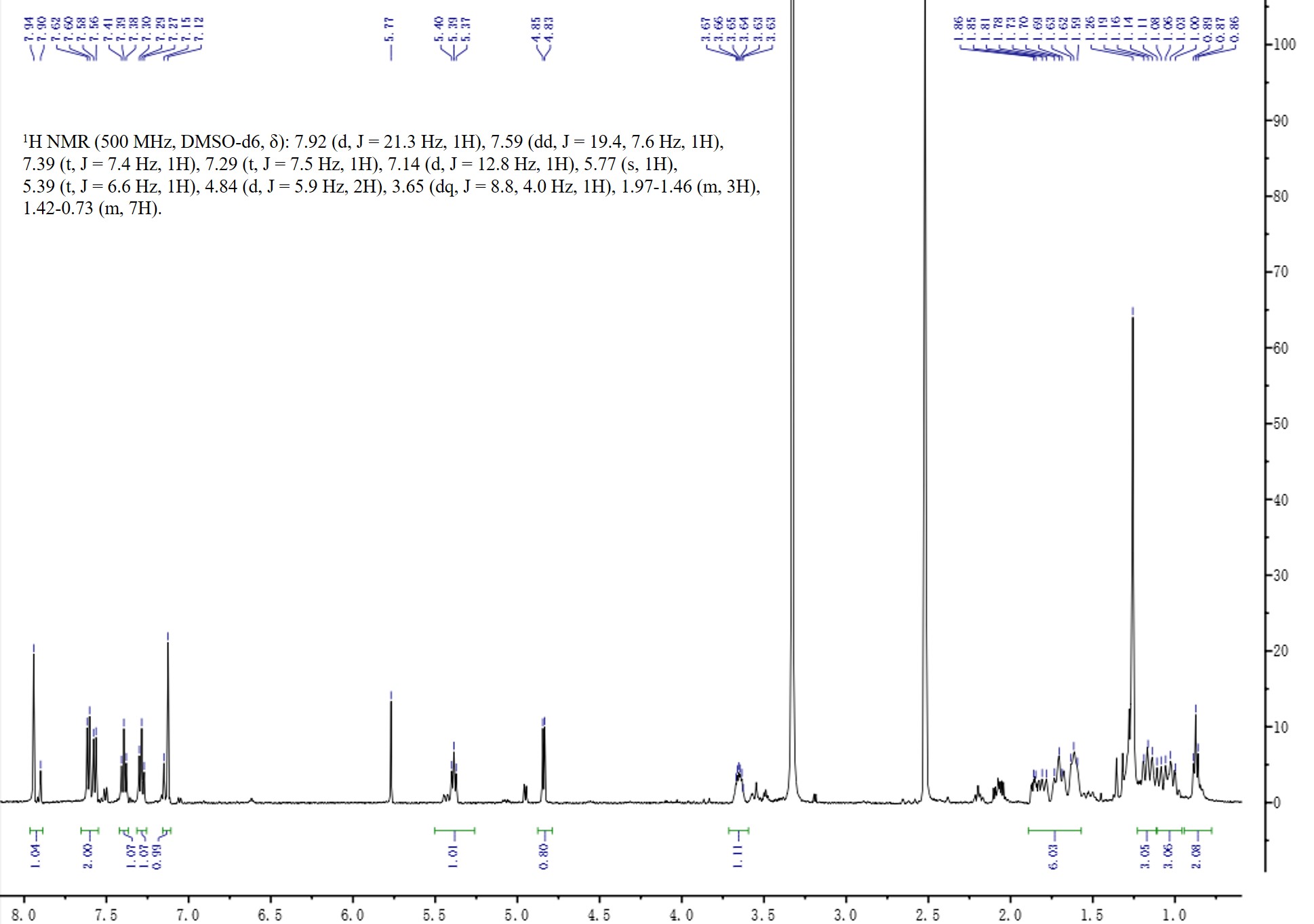
**

**Figure S4.** The ^1^H NMR of biotin-connected ICT. Methylene hydrogen (-CH_2_-) was detected in the biotin unit at δ 0.87, 1.04 and 1.26 ppm and methylene hydrogen (-CH_3_) in the ICT unit was at δ 1.57-1.89 ppm.

**Figure S5.** The synthetic route of BP_Avi._

**
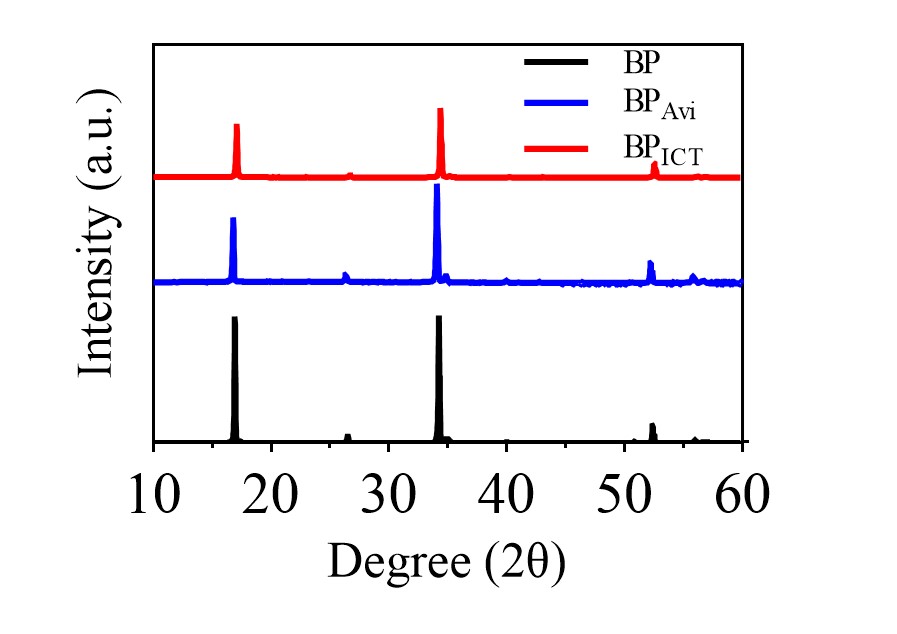
**

**Figure S6**. XRD of BP, BP_Avi_ and BP_ICT_.


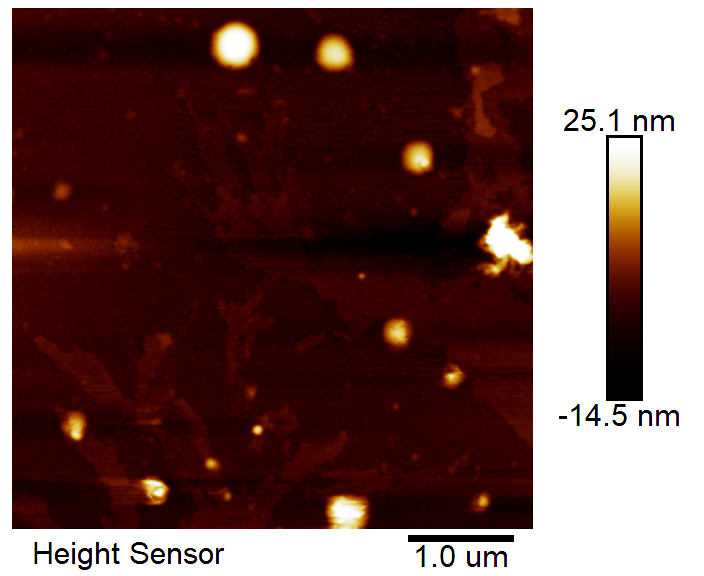


**Figure S7**. The AFM image of BP.


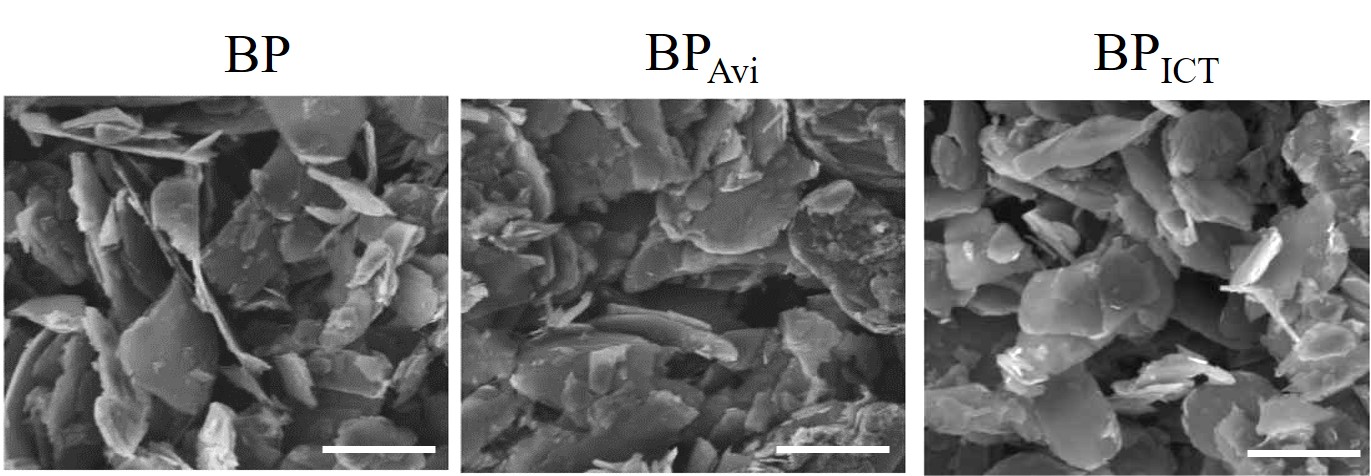


**Figure S8**. The SEM images of different formulations of BP. Scale bars: 100 nm.


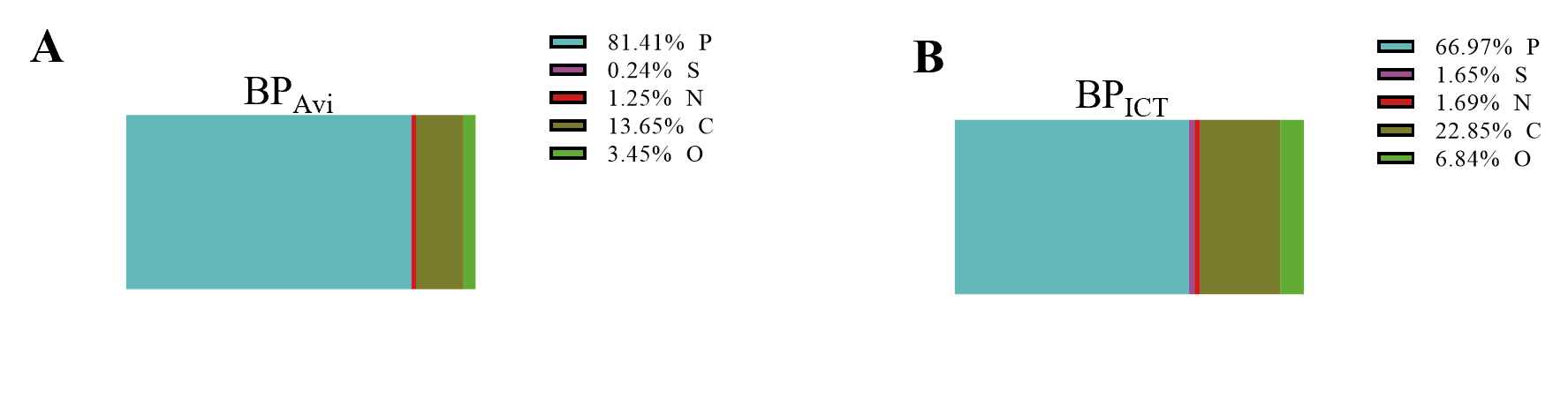


**Figure S9.** Quantitative results of elemental analysis of BP_Avi_ and BP_ICT_ (without SBF).


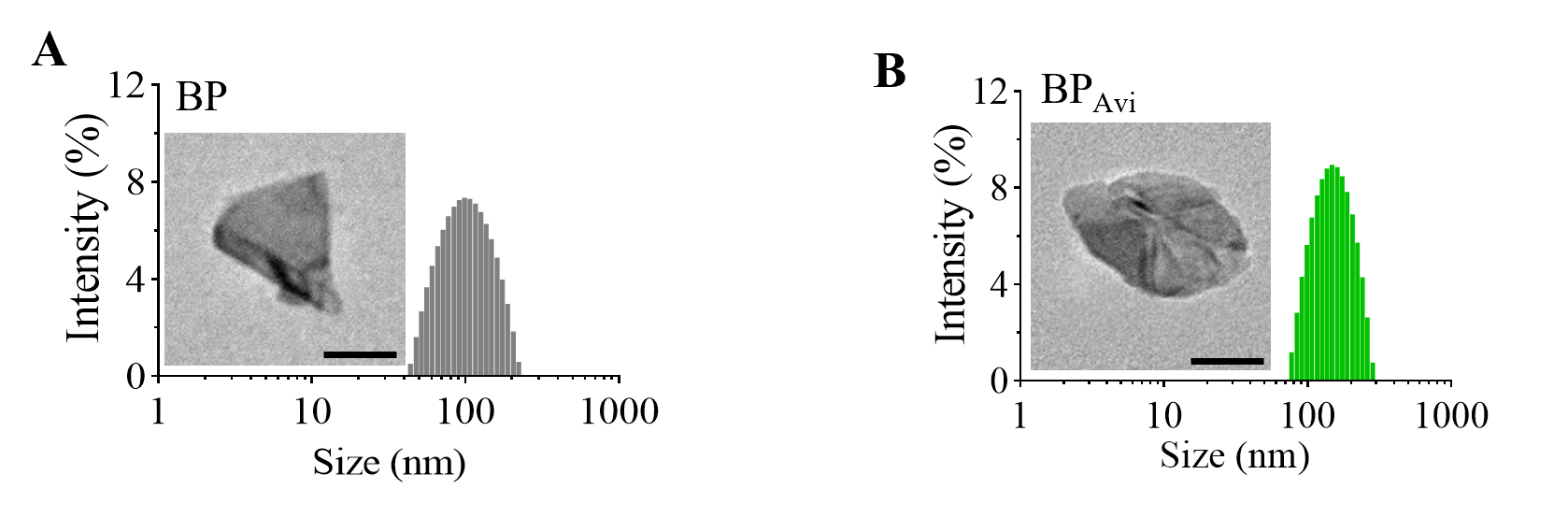


**Figure S10.** The size distribution and morphology of BP (A) and BP_Avi_ (B) determined by DLS and TEM, scale bars: 100 nm.

**
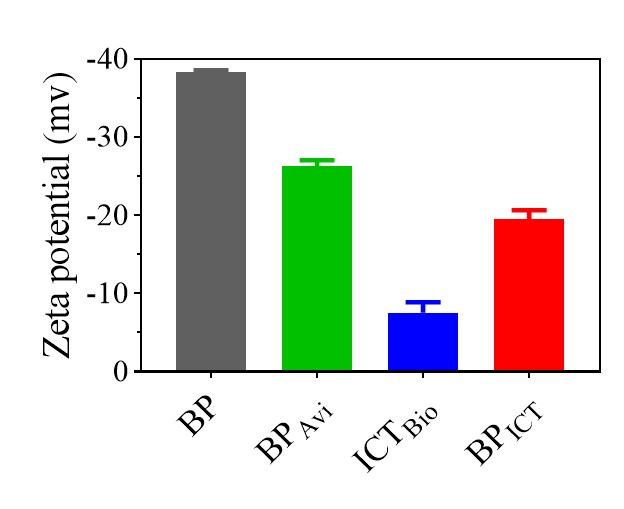
**

**Figure S11.** Zeta potential of BP, BP_Avi_, ICT_Bio_ and BP_ICT_.

**
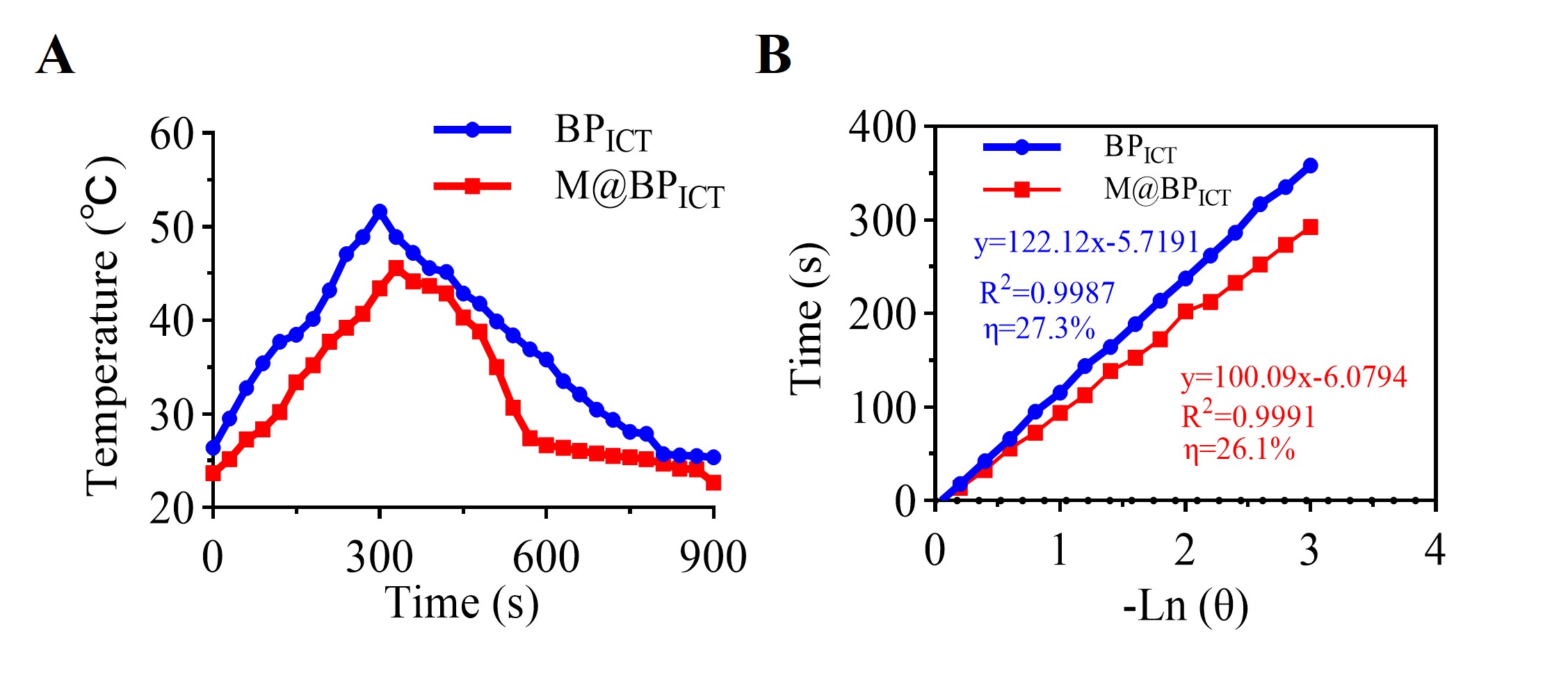
**

**Figure S12.** Photothermal conversion efficiency measurement. (A) Photothermal effect of the irradiation of the BP_ICT_ and M@BP_ICT_ (NIR: 808nm, 2.0 W cm^-2^, BP: 0.1 mg, 800 μL), in which the irradiation lasted for 5 min, and then the laser was shut off. (B) Photothermal conversion efficiency (ƞ) of BP_ICT_ and M@BP_ICT_ . Time constant for heat transfer from the system is determined by applying the linear time data from the cooling period (after 300 s) versus the negative natural logarithm of the driving force temperature, which is obtained from the cooling stage of (A).


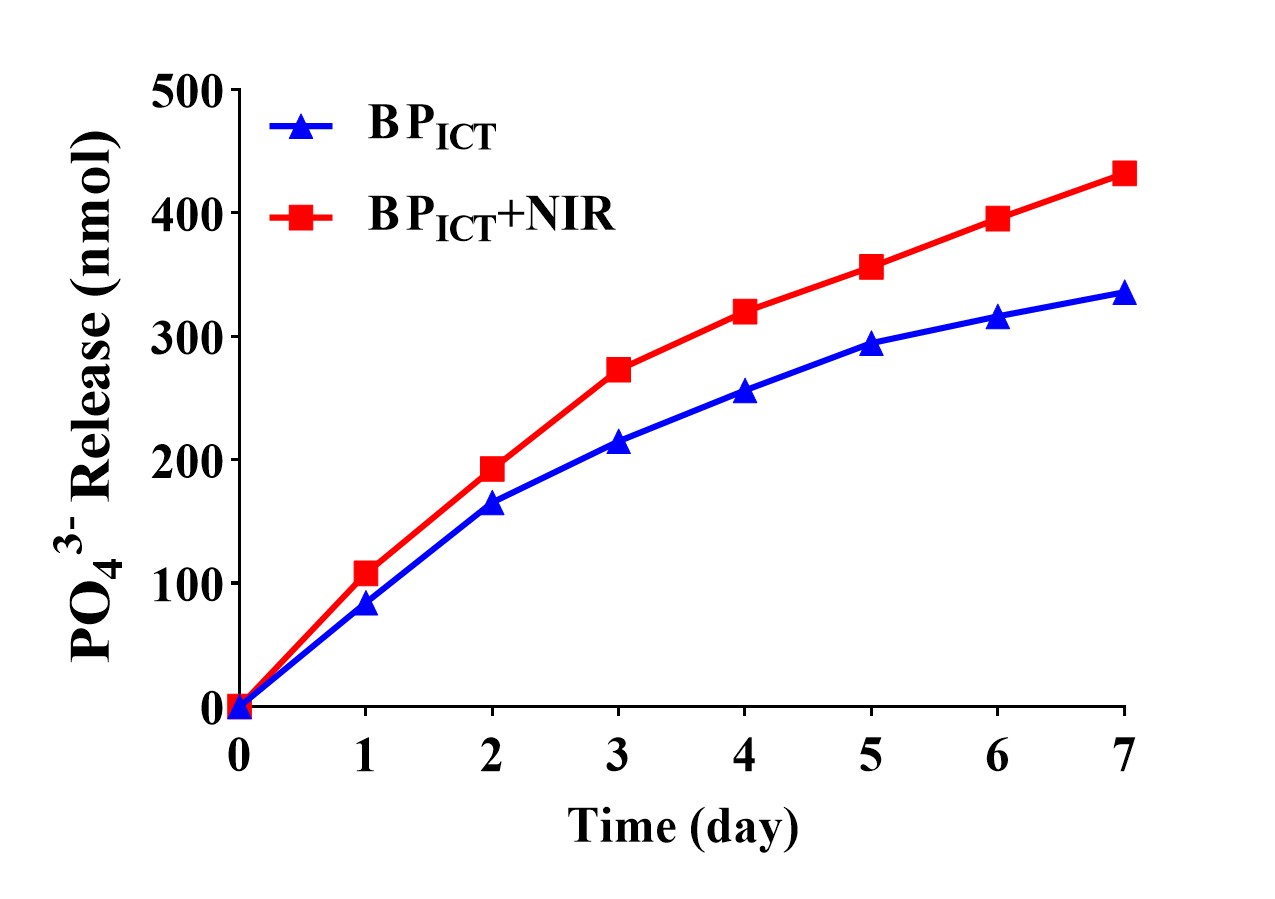


**Figure S13.** The cumulative release of P0_4_^3-^ from BP_ICT_ with or without NIR irradiation. (BP_ICT_, 1486.8 μg L^-1^, calculated by 1uM ICT. NIR irradiation condition: 808 nm, 1.0 W cm^-2^, 10 min per day.)


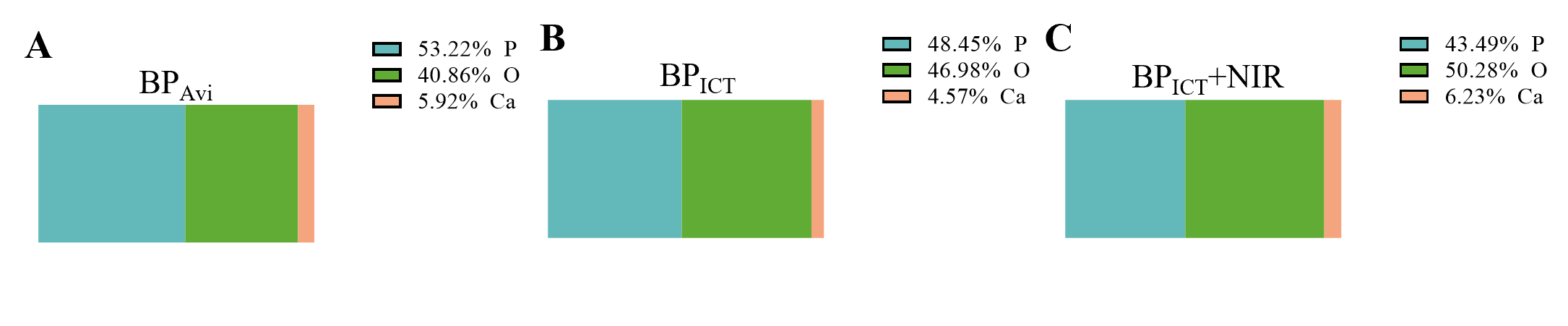


**Figure S14.** Quantitative results of elemental analysis of BP_Avi_ and BP_ICT_ (with SBF).


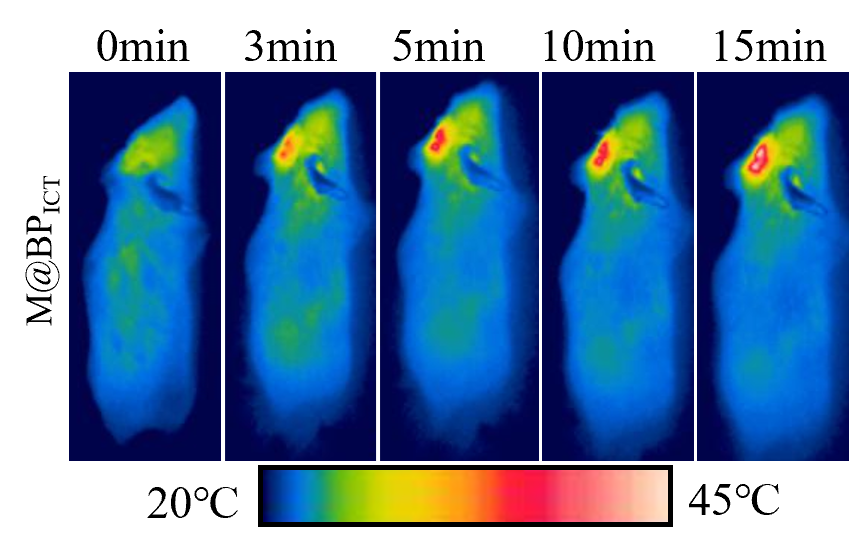


**Figure S15**. *In vivo* photothermal effect of M@BP_ICT_ was maintained for four weeks.


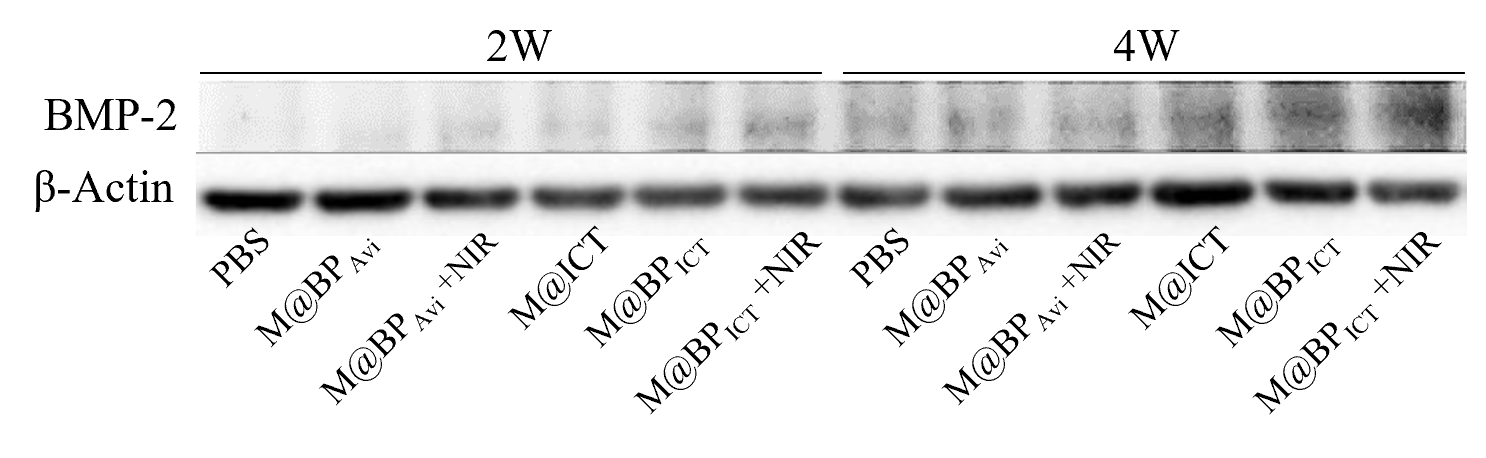


**Figure S16.** Representative western blot images of BMP-2.


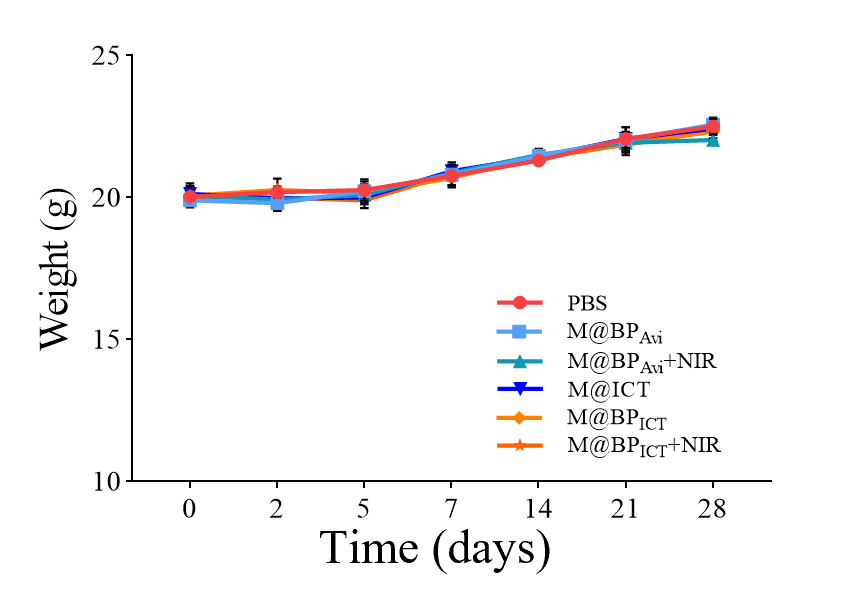


**Figure S17**. The body weight of mice with different treatments.


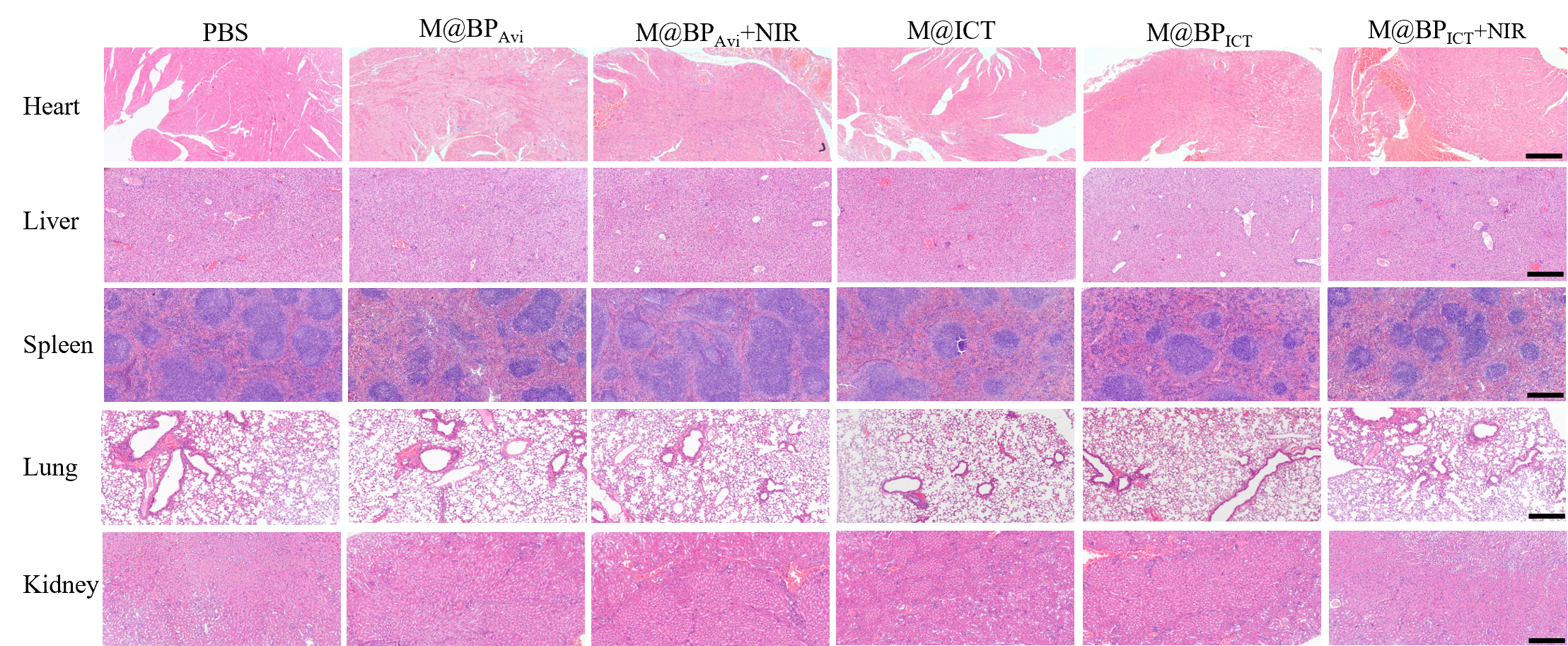


**Figure S18.** Representative H&E staining images of major organs including heart, liver, spleen, lung, and kidney collected from different groups. Scale bars: 200 μm.

**Table S1.** The Primer sequences used in this study.

| Gene name | Forward primer sequence (5’-3’) | Reverse primer sequence (5’-3’) |
| --- | --- | --- |
| BMP-2 | GTCTTCTAGTGTTGCTGCTTCCC | TCTCTGCTTCAGGCCAAACAT |
| Runx2 | GGAACCAAGAAGGCACAGACAG | TGTCTGCCTGGGATCTGTAATCT |
| Mrpl49 | CCAGAGCCACCAAAACATAAACA | GGTTGCCATGTGTGATCTCCTTA |
| Inhba | ACGGGTATGTGGAGATAGAGGAC | CAGACGGATGGTGACTTTGGT |
| Txnip | CCAGCCTACAGGTGAGAACGA | CTGGTGCCATTAAGTCAGGAGTAT |
| β-actin | TGCTATGTTGCCCTAGACTTCG | GTTGGCATAGAGGTCTTTACGG |
